# Update of the Situation-specific Theory for health management in heart failure: Delphi study*

**DOI:** 10.1590/1518-8345.7663.4554

**Published:** 2025-05-19

**Authors:** Gisele Saraiva Bispo Hirano, Viviane Martins da Silva, Alba Lucia Bottura Leite de Barros

**Affiliations:** 1 Centro Universitário das Faculdades Metropolitanas Unidas, São Paulo, SP, Brazil; 2 Universidade Federal do Ceará, Departamento de Enfermagem, Fortaleza, CE, Brazil; 3 Universidade Federal de São Paulo, Escola Paulista de Enfermagem, São Paulo, SP, Brazil

**Keywords:** Nursing Theory, Heart Failure, Standardized Nursing Terminology, Self-care, Delphi Technique, Nursing

## Abstract

to describe the process of analyzing the intrinsic elements of the Situation-specific Theory for health control in heart failure and present its respective update.

analysis of the intrinsic elements of the theory using the Delphi technique. Nurses whose expertise involved knowledge in Cardiology Nursing, Nursing theories, heart failure and/or Nursing terminology were selected.

the Theory was analyzed by 15 experts in the first round and 14 in the second round; all items evaluated obtained agreement greater than 80% regarding their adequacy, after two rounds of analysis. The theory maintained its original structure; however, the relationship between the factors that influence the health control of individuals with heart failure and the pictogram were updated.

the updated theory offers a better understanding of the factors that can influence the health control of individuals with heart failure and, although the focus of the theory is in the outpatient setting, it assumes the possibility of its application in other settings. The Delphi technique proved to be useful for theoretical validation, considering the specificity of the theme; however, the response time of the experts can be slow, which tends to impact the time to complete the study.

## Highlights

**(1)** Use of Delphi for internal validation of Situation-specific Theory. **(2)** Description of the characteristics of a Situation-specific Theory in nursing. **(3)** Innovation with the use of standardized languages in theory construction. **(4)** Advances in knowledge about health control in heart failure. **(5)** Improvement of nursing care for patients with heart failure.

## Introdução

Over the last century, nursing has evolved from an occupation to a profession, and with this, there has been a growing interest in building its own body of knowledge, abandoning empiricism to base clinical practice on scientific evidence. With the scientificization of the profession, nursing theories began to be developed with the intention of better understanding the phenomena and human responses that are of interest to nursing^(1-2)^. Nursing theories consist of a set of concepts and propositions that help to explain, predict and prescribe nursing care, that is, they underpin nursing knowledge and practice, and are therefore important for the development of the profession^(1)^.

Nursing theories can be classified, according to their level of abstraction, into grand theories (more abstract), mid-range theories (intermediate level of abstraction) and situation-specific theories (less abstract), or practical theories, depending on the literature consulted. The term “situation-specific theories” (SST) was first used in 1999 by Im and Meleis, when describing theories that were less abstract than mid-range theories and aimed at specific populations and situations that could be more easily applied in nursing clinical practice^(3)^. The properties of a SST, described by Im and Meleis, are: low level of abstraction; reflection on a specific nursing phenomenon, context, readily accessible connection to nursing research and practice, reflection of diversities in the nursing phenomenon, limited generalization^(3)^.

To determine the relevance and quality of a theory, it is suggested that it be subjected to an evaluation of its intrinsic elements or to clinical validation, based on empirical testing^(4)^. The evaluation of its elements should determine whether the epistemological approach is in accordance with what is expected, based on the reference used. Due to the specificity of the topic, submitting a nursing theory to an evaluation of its intrinsic elements can be a difficult task, since evaluators need to have high expertise for this purpose. Therefore, it is necessary to choose evaluation methods that are not only reliable, but also viable from a methodological point of view.

The evaluation of a theory, also called validation, is the main method for verifying its quality and suitability to pre-established criteria, in addition to highlighting the need for improvements to facilitate its use. Such validation can be from an internal perspective, which verifies the suitability of its intrinsic elements, or external, through empirical tests and several authors can be used as a guide^(5)^, as well as different structures can be used for this type of analysis^(6-7)^. In the case of situation-specific theories, the structure that describes the characteristics of this particular type of theory in more detail can be used as a guide^(3,8)^.

In 2023, the Situation-Specific Theory for health management in heart failure (HF) was published, a SST whose objective is to describe how the health management of patients with HF occurs and the factors that interfere in this control, using concepts from the standardized languages NANDA (NANDA-International), NIC (Nursing Interventions Classification) and NOC (Nursing Outcomes Classification) and Orem’s Self-Care Deficit Theory^(9)^. HF is a chronic, progressive disease, with symptoms and signs that result from any structural or functional impairment of blood filling or ejection by the ventricles^(10)^. Its treatment involves multidisciplinary monitoring, use of polypharmacy and frequent use of devices to control arrhythmias or ventricular assistance, which requires the individual to have good adherence to therapy, in addition to recognizing the symptoms of disease decompensation^(11)^. The central concept of the aforementioned SST was “health control”, considering that, despite the importance of good health control to reduce hospitalizations and improve quality of life, the literature shows that such individuals have difficulty in adequately managing their health^(9)^. This theory was constructed between 2019 and 2020 and, understanding that a SST should present the structure proposed by the literature^(3)^, which includes being easily applicable to clinical practice, it was decided to submit it to evaluation by specialists to assess its adequacy to the SST criteria described in the literature, in addition to other criteria related to nursing theories as a whole^(3,7)^.

Therefore, the objective of this study is to describe the process of analyzing the intrinsic elements of the Situation-specific Theory for health control in heart failure and present its respective update.

## Method

### Study design

To perform the analysis of the constructed theory, the Delphi technique was applied, a tool whose objective is to obtain a consensus of experts, through a series of questionnaires, about a complex topic^(12)^. The choice of this method was due to the scarcity of metatheorists, in the Brazilian scenario, who could contribute to the evaluation of this Theory and, as suggested by the literature, obtaining a consensus of a small group of experts can be useful to reach consistent results, although its use in the validation of nursing theories is still modest^(5)^. According to the literature, there is no consensus on the minimum number of individuals to be chosen as experts or the criteria that should be applied for their selection, which should be at the discretion of the researchers^(12)^. Similar studies used three to seven experts^(13-14)^, and for this study, the authors agreed that a minimum of 12 experts would be necessary, considering the loss of experts in each round^(15)^. To present the stages of the study, the SQUIRE checklist was used^(16)^.

### Expert selection criteria

**Figure 1 -:**
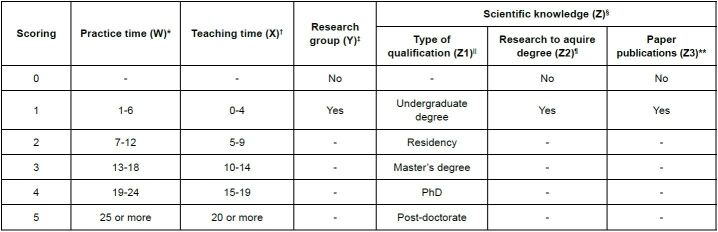
Parameters for the classification of experts according to the level of expertise of Benner, Tanner and Chesla. São Paulo, Brazil, 2022

The experts were recruited by combining data from our research experience and by searching the Lattes Platform of the National Council for Scientific and Technological Development (CNPq) website. Nurses whose expertise involved knowledge in Cardiology Nursing, Nursing theories, heart failure and/or Nursing terminology were selected. During the selection of experts, we considered not only their knowledge of the construction of theories themselves, but also their knowledge of the population studied and the structure that supported the construction of their concepts (standardized nursing language systems). The experts were characterized according to their age and gender, and for their characterization, in relation to their academic training and clinical and teaching experience, the criteria established by Benner, Tanner and Chesla^(17)^ in 2009 for the selection and classification of experts were adopted: practical experience determined by the time of experience in Cardiology; and academic experience determined by participation in research groups on: Cardiology, standardized languages and/or Nursing theories, as well as the production of knowledge and the publication of articles/scientific papers on the mentioned themes. It is worth noting that the intervals constructed for the practice and teaching times were established by the authors themselves based on the minimum and maximum complete years presented by the experts. The “qualification” criterion received scores from 1 to 5 (1 – undergraduate degree; 2 – residency; 3 – master’s degree; 4 – PhD; 5 - post-doctorate fellowship). For the “research” requirement for the degree and the publication of articles on the suggested themes, scores of 0 were assigned for absence and 1 for presence. The level of expertise was calculated by the simple average of the scores obtained, as shown in Figure 1.

*W = Years (range constructed from minimum and maximum practice times); ^†^X = Years (range constructed from minimum and maximum teaching experience time); ^‡^Y = Participation in research group on the topic; ^§^Z = Training and scientific production on the topic; ^||^Z1 = Academic training; ^¶^Z2 = Dissertation or thesis on the topic; **Z3 = Published articles on the topic

**Figure 2 -:**
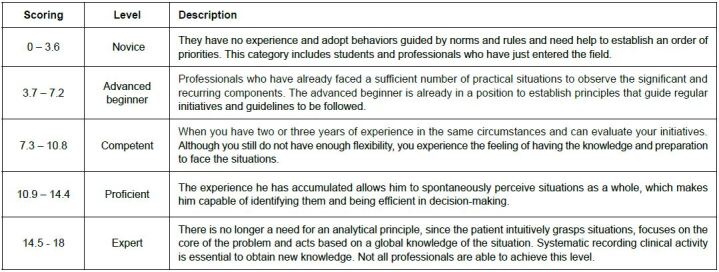
Level of expertise of judges. São Paulo, Brazil, 2022

These parameters led to the classification of experts according to their level of expertise^(17)^, as described in Figure 2.

The invitation, sent via email, included a description of the study objectives and the Informed Consent Form (ICF). After accepting and signing the ICF, the selected experts received the theory in full and completed the analysis instrument. Data collection took place between August 2021 and April 2022.

### Elements evaluated by experts

The elements contained in the analysis instrument included those suggested by the literature^(3,7)^.

According to the literature, situation-specific theories should contain the following properties^(3)^:

- *Low level of abstraction*: less abstract than mid-range theories and more abstract than experience reports, guidelines, protocols, care plans and action plans;

- *Reflection of a specific nursing phenomenon*: reflects upon a particular field of practice; aims to develop and understand specific populations and provides frameworks or projects for action;

- *Context*: should address the social, historical and cultural context; its scope is limited to specific situations or populations;

- *Readily accessible connection to nursing research and practice*: should provide a link between theory, research and practice;

- *Reflection of diversities in the nursing phenomenon*: should include diversities, which address not only cultural and political aspects, but also gender, religiosity, education, among other aspects;

- *Limited generalization*: by reflecting diversities and complexities of a specific nursing phenomenon, generalization is limited;

A theory can still be evaluated with respect to its significance and pragmatic adequacy and its elements with respect to their internal consistency, parsimony, empirical adequacy and pragmatic adequacy^(7)^:

- *Significance*: justifies the importance of the theory for the Nursing discipline;

- *Internal consistency*: assesses whether the concepts of the theory present clarity and semantic consistency;

- *Parsimony*: this criterion requires that a theory be stated in the most economical way possible, without oversimplifying the phenomena of interest, i.e., it is best that few concepts and propositions are needed to fully explain the phenomena of the theory;

- *Pragmatic adequacy*: usefulness of the theory in practice. When reviewing the descriptions of the theory, it will be analyzed whether it can actually be used in the real world, in clinical practice, i.e., its viability is assessed.

The empirical adequacy, also suggested by the framework used^(7)^, was not evaluated because it analyzes whether the statements made by the theory are consistent with empirical evidence found in studies that use it as a basis for research, which was not possible, since it was a newly constructed theory.

The properties of the SST and the criteria of significance and pragmatic adequacy were applied to the theory as a whole; the concepts were evaluated according to internal consistency, the propositions and assumptions were evaluated for parsimony.

The analysis of the pictogram was performed using the following criteria^(6)^:

-Graphical and visual representation of the theory;

-Logical representation;

-Clarity

Each item was evaluated in relation to frequency and proportion of agreement, with items that obtained agreement equal to or greater than 80% among the experts, therefore, the item could be considered adequate^(18)^.

### 1^st^ round

Electronic submission: document with a brief description of the theory construction method, the preliminary version of the constructed theory and an instrument in which the experts were asked to indicate their degree of agreement regarding the properties of the theory, in addition to its significance, internal consistency, parsimony and pragmatic adequacy, and specific characteristics of the pictogram. After 15 days, a reminder was sent to non-respondents and a further 15 days were waited. Due to the low number of responses from the experts, after the second reminder, a new e-mail was sent, requesting that they fill in the response. Some experts showed interest in participating after the second reminder, but did not respond within the pre-established deadline. In these cases, telephone contact was made and a further 20 days were waited. Although, as mentioned, there is no minimum number of experts to conduct Delphi, a minimum number of 12 experts was considered ideal to analyze this study. In this way, the response deadline was postponed in order to obtain the minimum desired quantity, resulting in a total deadline of four months. After this deadline, the analysis process was finalized to collect and analyze the data from the first round.

The instrument sent to the experts was composed of two parts: part A: demographic, academic and professional characterization; part B: questions about the analysis of the theory itself. This instrument proposed an analysis of the theory as a whole, evaluating: the properties of the theory, the internal consistency of the concepts, parsimony and pragmatic adequacy, in addition to specific elements such as: clarity, relevance and relevance to the pictogram. For each item, the subjects were asked to indicate the degree of agreement on a five-point Likert scale ranging from “completely disagree” to “completely agree”, in addition to an open space for suggestions for changes and improvements for each item evaluated. The data collected in the first round were tabulated in Excel spreadsheets and analyzed using descriptive statistics using the R software. Each item was evaluated in relation to frequency and proportion of agreement, with the item being considered adequate if it obtained an agreement equal to or greater than 80% among the experts. If the expert indicated “I neither agree nor disagree” (3 points), “I slightly disagree” (2 points) or “I completely disagree” (1 point), they were asked to offer suggestions regarding the necessary modifications to the theory. For each proportion, 95% confidence intervals were calculated using the Wald method. The confidence interval was used to establish with 95% confidence that the estimated proportion found was statistically equal to or greater than the established cutoff point (80%).

### 2^nd^ round

The second round took place after the changes suggested in the first round had been implemented and was implemented in a similar way to the previous one. In the second round, the experts were presented with the theory and its modifications, the instrument analysis, and general feedback on the changes made based on the suggestions made in the first round, the proportions of agreement found for the elements, and the proposed suggestions. All material was sent to the experts via email, with reminders every 15 days. After three months, in order to obtain the largest number of participating experts, a reminder was sent by telephone to those who had not responded to the sent emails. This round took place between December 2021 and April 2022.

### Ethical aspects

This study was approved by the Ethics and Research Committee (CEP) of the Federal University of São Paulo, under the number 3,903,496. All ethical precepts set in Resolution 466/2012^(19)^ were met. An invitation letter was sent electronically (through e-mail) to the selected experts together with the Informed Consent Form (ICF). The ICF presented the objectives of the research, its voluntary aspect and the data collection procedures. Researchers who returned the ICF signed were considered accepted to participate in the research.

## Results

To perform the theory analysis, 22 nurses with knowledge in Cardiology Nursing, Nursing theories, heart failure and/or Nursing terminology were selected. After the invitation and reminders sent every 15 days, over a period of one month, only two experts had responded to the instrument analysis. Thus, new reminders were sent over a period of four months with 15-day intervals, making it possible to obtain responses from a total of 15 experts for the first round of analysis.

### Characterization of experts

The experts selected to analyze the preliminary version of the constructed theory were nurses with extensive knowledge and experience in Cardiology, standardized languages and/or Nursing theories. The group consisted of two men and 13 women, aged from 31 to 62 years old. To characterize the experts according to their academic training and clinical and teaching experience, previously established criteria were adopted^(11)^, and the level of expertise was calculated by the simple average of the scores obtained.

Based on this classification, it was identified that the experts who performed the analysis were mostly “competent” (eight) or “proficient” (six), with only one being classified as a proper “expert”, which denotes a significant level of expertise of the experts involved in the analysis of this study.

### Results from the 1^st^ round of analysis

After completing the first part of the instrument analysis, the experts indicated their level of agreement with the items described. The complete instrument contained 25 items, with items 1 to 19 addressing the analysis of the theory (theory as a whole, concepts, assumptions and propositions) and items 20 to 25 related to the analysis of the pictogram. For the analysis of agreement, the responses “I agree” and “I completely agree” were compiled, as well as the responses “I disagree” and “I completely disagree”. Neutral responses (I neither agree nor disagree) were not considered for the purpose of analysis. Each item was evaluated in relation to the frequency and proportion of agreement, with items that obtained agreement equal to or greater than 80% among the experts being considered adequate, as shown in Table 1.

**Table 1 -:** Results of the experts’ evaluation in the first round of item evaluation, according to the percentage of experts who said they agreed with the items (n = 15*). São Paulo, Brazil, 2022

**Items**	**n** ^†^	**%** ^‡^	**CI 95%** ^§^
Item 1 - use of non-abstract concepts to explain the health management of patients with heart failure.	15	100.0	74.6 – 100.0
Item 2 - explicit presentation of the specific situation addressed (health management of individuals with heart failure).	15	100.0	74.6 – 100.0
Item 3 - provision of a structure that specifically represents the health management of patients with heart failure.	15	100.0	74.6 – 100.0
Item 4 - integrative approach to theory construction.	12	80.0	51.4 – 94.7
Item 5 - specificity of the theory to the outpatient population with heart failure.	13	86.7	58.4 – 97.6
Item 6 - possibility of connecting the propositions between theory and practice	13	92.9	64.2 – 99.6
Item 7 - provides directions for its use in research.	14	100.0	73.2 – 100.0
Item 8 - allows the establishment of guidelines for practice	14	100.0	73.2 – 100.0
Item 9 - incorporation of cultural, economic and clinical aspects of the patient with HF ^||^ .	13	86.7	58.4 – 97.6
Item 10 – incorporation of barriers and difficulties encountered by the population with heart failure in controlling their health	15	100.0	74.6 – 100.0
Item 11- filling existing knowledge gap.	14	100.0	73.2 – 100.0
Item 12 – ampliação de um conhecimento existente.	14	93.3	66.0 – 99.6
Item 13 – explicit presentation of the conceptual model from which the theory was derived	15	100.0	74.6 – 100.0
Item 14 - possibility of identifying the interpersonal and psychomotor skills of nurses that are necessary for its application.	15	100.0	74.6 – 100.0
Item 15 - feasibility of implementing clinical protocols derived from theory	15	100.0	74.6 – 100.0
Item 16 – possibility of achieving good results with the Nursing actions proposed by the theory	14	100.0	73.2 – 100.0
Item 17 - the phenomenon studied (health control) is adequately expressed by the concepts	14	93.3	66.0 – 99.6
Item 18- possibility of categorizing and interpreting the phenomenon covered by the theory through concepts	13	86.7	58.4 – 97.6
Item 19 – approach the content in a clear and concise manner	15	100.0	74.6 – 100.0
Item 20 – adequate graphic and visual representation by pictogram	14	93.3	66.0 – 99.6
Item 21 - possibility of better understanding the concepts of the theory through the pictogram	13	92.9	64.2 – 99.6
Item 22 – representation of the main concepts, in the pictogram	14	93.3	66.0 – 99.6
Item 23 – relevance of the pictogram to the theory as a whole	15	100.0	74.6 – 100.0
Item 24 - logical presentation of the relationship between concepts in the pictogram	13	86.7	58.4 – 97.6
Item 25 – possibility of better understanding the text and the relationships between concepts through the pictogram	15	100.0	74.6 – 100.0

*Total number of experts; ^†^n = Total number of experts who responded that they agreed with the item evaluated; ^‡^% = Proportion of experts who agreed with the item evaluated; ^§^CI95% = 95% confidence interval; ^||^HF = Heart failure. Note: Items with n<15 were those that did not receive a response and/or the response “I neither agree nor disagree”

**Figure 3 -:**
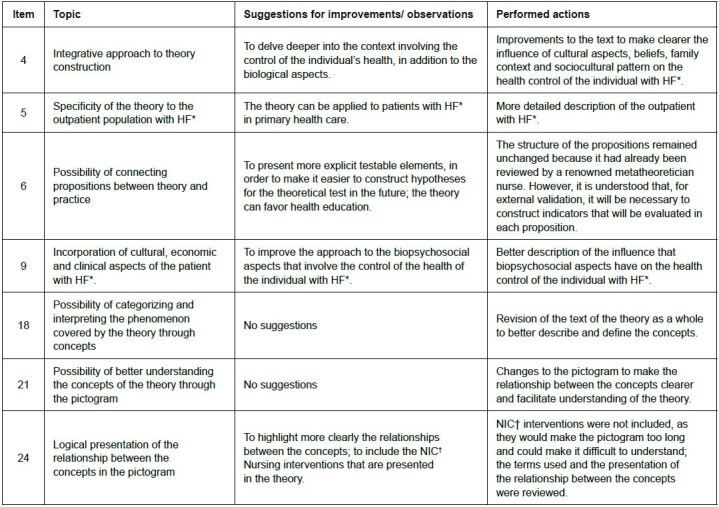
Items evaluated that presented a wide confidence interval, suggestions for improvements in the theory and actions taken by the author. São Paulo, Brazil, 2022

Although agreement of over 80% was obtained in all items analyzed in the first round, some items presented a very wide confidence interval and, for this reason, improvements were implemented in the body of the theory, guided by the suggestions proposed by the experts. A summary of the suggestions for improvements that were proposed for these items is presented in Figure 3.

*HF = Heart failure; ^†^NIC = Nursing Interventions Classification

### Results of the 2^nd^ round of analysis

After the changes suggested in the first round had been made, the updated text of the SST was sent again to the same experts from the 1^st^ round, together with a report informing the results of the first round and the changes made to the text and pictogram, according to the suggestions received.

The revised theory was sent in full to the experts, with the changes in the text highlighted in blue, together with a new instrument analysis, this time with only the items that had been revised, to be answered, totaling eight items. Items 1 to 5 referred to the theory and its elements, while items 6 to 8 referred to the pictogram. The time between sending the first email, in this second phase, with the request to complete the analysis and the responses from the last expert was approximately three months, and in the 2^nd^ round, 14 experts responded, one less than in the 1^st^ round.

Based on the results of this second round, 100% agreement was obtained in almost all items, as well as a reduction in the confidence interval. The items that presented agreement below 100% were: item 2, regarding the possibility of generalizing the theory (71.4%), and item 8 (92.9%), regarding the clarity of the pictogram.

Based on the suggestions that had already been given in the first round, the authors assume that, although the theory was developed to be specific to the outpatient population with HF, it can also be applied in the primary health care sphere, since the conditioning factors that affect the health control of the individual treated at this level of health care may be similar, making it useful in this scenario as well. This hypothesis may be confirmed, or refuted, after the future development of indicators that allow the clinical validation of the theory. Regarding the pictogram, since the agreement obtained was above 80%, the version sent for analysis was considered appropriate. Therefore, the version analyzed by the experts in the second round of analysis, due to high levels of agreement in the elements evaluated, was considered adequate and constituted the final version of the theory.

## Discussion

Based on the analysis by experts, the SST for health control in HF could have its concepts reviewed and clarified, and its interrelations presented more clearly in the pictogram. This theory was initially constructed with the objective of describing the health control of people with HF in outpatient care. However, after the analysis by experts, it is suggested that it can also be useful in the context of primary health care, as it is often the first point of contact between the user and the health system and is an environment that can promote health education and improve adherence to treatment.

It is also considered that the nurse can educate and guide the patient in the most diverse care settings, and although the outpatient setting or at the primary care unit can be more conducive for this to occur, future testing of the theory should be conducted in other settings.

### The revised theory

It is understood that individuals with HF who are able to control their health use their free will to perform actions in order to maintain their life, health and well-being. According to the literature^(20)^, this process involves actions to maintain physiological stability, recognition of symptoms that indicate clinical worsening and appropriate actions when worsening symptoms are observed. The elements that influence the control of health of individuals with HF are diverse and it is important that they are known by the multidisciplinary team so that appropriate planning of the interventions to be implemented can be carried out. For this reason, individual`s health should be understood beyond the biological context, but also including the context in which they are inserted, such as their family structure, cultural and biopsychosocial aspects, as recommended by the principle of Comprehensiveness of the Unified Health System (SUS)^(21)^.

For the individual to be able to control their health, they must first want to commit to achieving this control. In order to achieve health control, the individual must strive to develop specific skills and abilities, which will be demonstrated through health control behaviors.

The elements together can act favorably or unfavorably in the adequate control of the health status of the individual with HF are called: basic conditioning factors. Some characteristics make individuals more susceptible to not controlling their health satisfactorily, categorizing them as a “population at risk” of presenting ineffective health control. Some elements, such as the presence of comorbidities, high functional class, medical devices related to HF and complex drug treatment, are understood as “associated conditions” and can also lead the individual to present ineffective health control.

In the case of inefficient health control, the individual will present the ND Ineffective Health Control [00078], evidencing the need for Nursing interventions. In the case of efficient interventions, it is expected that health control will be achieved, and in this case, new educational interventions and those that reinforce positive behavior should be implemented. Thus, the more elements that negatively influence the individual’s health control, the greater the demand for nursing interventions to help them achieve this control. Individual demands for interventions that stimulate or lead to health control should be investigated in order to identify the key points to be addressed by the nurse in developing their therapeutic plan. The proposed nursing activities comprise the NIC interventions Cardiac care [4040], Health education [5510] and Teaching: disease process [5602]. The nurse should act in the guidance, monitoring and encouragement of behaviors aimed at health control, and, to this end, can use as a guide the NOC outcome indicators Self-control of heart disease [1617] and Adherence behavior [1600].

In the construction of the first version of the theory, after establishing the theoretical concepts and their interrelations, its assumptions and propositions were established^(7)^. These components, after analysis by the experts, remained unchanged and are presented below:

### Assumptions

- Committing to health control in HF requires the ability to take care of oneself;

- The ability to control health can be affected by factors that are inherent to the individual, such as beliefs and values, in addition to those related to the complexity of the health system;

- Commitment to health care and efforts to control health in HF are influenced by factors that are intrinsic and extrinsic to the individual;

- Commitment to health care and efforts to control health in HF enable the individual to know what to do and how to act when symptoms of decompensation occur;

- Nurses promote ways to assist individuals with HF, according to their needs and difficulties in controlling their health;

- Nursing is one of the health services that is able to assist individuals with HF to control their health satisfactorily.

### Propositions

- Changes in health control of individuals with HF are related to changes in behaviors that can be expressed by the NOC outcome indicators Adherence Behavior [1600] and Heart Disease Self-Management [1617]^(22-24)^;

- Individuals with HF with Ineffective Health Control may express the following characteristics: difficulty with the prescribed regimen, ineffective daily life choices to achieve health goals, failure to act to reduce risk factors, failure to include the treatment regimen in daily life^(25-26)^;

- The commitment to health control of individuals with HF can be influenced by conditioning factors, such as: sociocultural orientation^(27)^, health status, health system factors, family systems^(25-26,28)^, living standards, availability of resources and adequacy^(25,28-29)^, environmental factors, perceived barriers and benefits, decision-making conflict, insufficient knowledge^(30)^, feeling of helplessness, perceived susceptibility^(31)^, excessive demands, difficulty in controlling a complex treatment regimen^(32)^.

**Figure 4 -:**
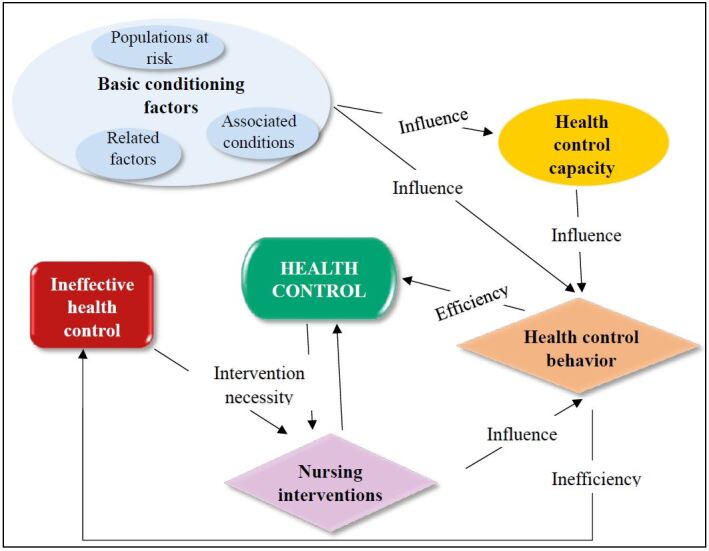
Pictogram involving metaparadigms, central and peripheral concepts of the Situation-specific Theory for health control in heart failure. São Paulo, Brazil, 2022

The pictogram that expresses the relationship between the concepts was updated, according to the proposed suggestions and expresses the interaction between the concepts of this SST (Figure 4).

Despite the importance of good health management to improve quality of life and reduce hospitalizations, it is known that many patients with HF have the ND Ineffective Health Control. Several internal and external factors influence the acquisition of health control and, although not all of them are amenable to nursing interventions, their identification can facilitate the diagnostic process by the nurse.

By guiding the nurse in identifying both clinical aspects that indicate that the disease is not under control and environmental and individual factors that interfere with the individual’s ability to adequately control their health, this theory aims to contribute to the adoption of strategies that encourage individuals with HF to include the therapeutic regimen in their daily lives, improving their quality of life and survival.

Its propositions reflect the interactions between the established concepts and are subject to future testing with the population in question. To this end, the Nursing activities proposed in the NIC interventions Cardiac care [4040], Health education [5510] and Teaching: disease process [5602] and the NOC result indicators Self-management of heart disease [1617] and Adherence behavior [1600] may be used.

## Conclusion

The validation performed through the Delphi technique allowed the theory to be evaluated by experienced experts and its text and pictogram to be improved in order to more accurately reflect the phenomenon studied (health control) and facilitate its incorporation into the daily practice of nurses who care for individuals with HF, whether in outpatient or primary health care settings.

Validating a theory, whether from an internal perspective or from empirical testing, is not an easy task and requires, in addition to time and attention, great expertise in the method of theoretical construction and the topic addressed. The Delphi technique, although still little used for this purpose, has proven useful because it allows consensus to be reached without the need for a large number of experts, which is particularly advantageous in topics where there is a shortage of experts. In addition to the need to find professionals with the appropriate expertise to carry out the proposed validation, a major difficulty was the response time of the judges in each round, which impacted the time to complete the study. Furthermore, the analysis performed allowed us to confirm that the theory constructed presents the properties and characteristics expected of the SST.

After internal validation and updating, the SST for health control in HF offers a better understanding of the factors that can influence the health control of individuals with HF. As it is a theory based on Orem’s Self-Care Deficit Nursing Theory and uses concepts from the standardized languages NANDA, NIC and NOC, already widely used in clinical practice, it is expected that it can contribute to eliminating the dichotomy between thought and action, so that the nurse in practice can clearly diagnose the aspects that involve the health control of their patients and how Nursing should act in caring for these individuals. It is hoped that, in the future, instruments will be constructed that allow the evaluation of the individual in a more concrete way and, for this purpose, the indicators of the NOC results “adherence behavior” and “self-control of heart disease” can be used as guides.

Considering that care for individuals with heart failure involves a multidisciplinary approach and that some factors that influence health control cannot be addressed exclusively by the Nursing field, it is believed that this theory may also be useful for other disciplines.
