# Supplementary material for: Update of the Situation-specific Theory for health management in heart failure: Delphi study
Source: Rev Lat Am Enfermagem. 2025 May 19;33:e4554. doi: 10.1590/1518-8345.7663.4554 (PMC12088657; doi:10.1590/1518-8345.7663.4554)
Supplement: Translation

# Translation: Atualização da Teoria de Situação-específica para o controle da saúde na insuficiência cardíaca: estudo Delphi*

**DOI:** 10.1590/1518-8345.7663.4555

**Authors:** Gisele Saraiva Bispo Hirano, Viviane Martins da Silva, Alba Lucia Bottura Leite de Barros

**Affiliations:** 1 Centro Universitário das Faculdades Metropolitanas Unidas, São Paulo, SP, Brasil; 2 Universidade Federal do Ceará, Departamento de Enfermagem, Fortaleza, CE, Brasil; 3 Universidade Federal de São Paulo, Escola Paulista de Enfermagem, São Paulo, SP, Brasil

**Keywords:** Teoria de Enfermagem, Insuficiência Cardíaca, Terminologia Padronizada em Enfermagem, Autocuidado, Técnica Delphi, Enfermagem

## Abstract

descrever o processo de análise dos elementos intrínsecos da Teoria de Situação-específica para o controle da saúde na insuficiência cardíaca e apresentar sua respectiva atualização.

análise dos elementos intrínsecos da teoria utilizando técnica Delphi. Foram selecionados enfermeiros cuja expertise envolvia conhecimentos em Enfermagem em Cardiologia, teorias de Enfermagem, em insuficiência cardíaca e/ou terminologias de Enfermagem.

a Teoria foi analisada por 15 especialistas na primeira rodada e 14 na segunda rodada; todos os itens avaliados obtiveram concordância superior a 80% com relação à sua adequação, após duas rodadas de análise. A teoria manteve sua estrtutura original, no entanto, a relação entre os fatores que influenciam o controle da saúde do indivíduo com insuficiência cardíaca e o pictograma foram atualizados.

a teoria atualizada oferece melhor compreensão acerca dos fatores que podem influenciar o controle da saúde dos indivíduos com insuficiência cardíaca e, embora o foco da teoria seja o cenário ambulatorial, assume-se a possibilidade de sua aplicação em outros cenários. A técnica Delphi mostrou-se útil para a validação teórica, tendo-se em vista a especificidade do tema, contudo o tempo de resposta dos especialistas pode ser demorado, o que tende a impactar no tempo de conclusão do estudo.

## Destaques

**(1)** Utilização de Delphi para validação interna de Teoria de Situação-específica. **(2)** Descrição das características de uma Teoria de Situação-específica em enfermagem. **(3)** Inovação com o uso de linguagens padronizadas na construção de teoria. **(4)** Avanços no conhecimento sobre o controle da saúde na insuficiência cardíaca. **(5)** Aprimoramento da assistência de enfermagem aos indivíduos com insuficiência cardíaca.

## Introdução

Ao longo do último século, a enfermagem evoluiu de ocupação para profissão e, com isso, houve um interesse crescente em construir um corpo de conhecimento próprio, abandonando o empirismo para alicerçar a prática clínica em evidências científicas. Com a cientifização da profissão, começaram a ser desenvolvidas teorias de enfermagem na intenção de compreender melhor os fenômenos e as respostas humanas que são de interesse da enfermagem^(1-2)^. As teorias de enfermagem consistem em um conjunto de conceitos e proposições que ajudam a explicar, prever e prescrever cuidados de enfermagem, ou seja, alicerçam os saberes e a prática de enfermagem, sendo, portanto, importantes para o desenvolvimento da profissão^(1)^.

As teorias de enfermagem podem ser classificadas, de acordo com seu nível de abstração, em grandes teorias (mais abstratas), teorias de médio alcance (nível intermediário de abstração) e teorias de situação-específica (menos abstratas), ou teorias práticas, a depender da literatura consultada. O termo “teorias de situação-específica” (TSE) foi utilizado pela primeira vez em 1999, por Im e Meleis, ao descreverem teorias menos abstratas que teorias de médio alcance e voltadas a populações e situações específicas que pudessem ser mais facilmente aplicáveis na prática clínica do enfermeiro^(3)^. As propriedades de uma TSE, descritas por Im e Meleis, são: baixo nível de abstração; reflexão sobre um fenômeno específico de enfermagem, contexto, conexão prontamente acessível à pesquisa e à prática de enfermagem, reflexão de diversidades no fenômeno de enfermagem, generalização limitada^(3)^.

Para determinar a relevância e a qualidade de uma teoria, sugere-se que ela seja submetida a uma avaliação de seus elementos intrínsecos ou a uma validação clínica, a partir de teste empírico^(4)^. A avaliação de seus elementos deverá determinar se a abordagem epistemológica está de acordo com que é esperado, a partir da referência utilizada. Devido à especificidade do tema, submeter uma teoria de enfermagem a uma avaliação de seus elementos intrínsecos pode ser uma tarefa árdua, já que é necessário que os avaliadores possuam alta expertise para este fim. Dessa maneira, faz-se necessário a escolha de métodos de avaliação que sejam não somente confiáveis, mas também viáveis do ponto de vista metodológico.

A avaliação de uma teoria, também chamada de validação, é o principal método para verificar sua qualidade e adequação a critérios pré-estabelecidos, além de pontuar as necessidades de melhorias para facilitar seu uso. Tal validação pode ser da perspectiva interna, isto é, a que verifica a adequação de seus elementos intrínsecos, ou externa, por meio de testes empíricos nos quais vários autores podem ser utilizados como guia^(5)^, bem como diferentes estruturas podem ser utilizadas para esse tipo de análise^(6-7)^. No caso de teorias de situação-específica, pode-se utilizar como guia a estrutura que descreve de modo mais detalhado as características desse tipo de teoria em particular^(3,8)^.

Em 2023, foi publicada a Teoria de Situação-específica para o controle da saúde na insuficiência cardíaca (IC), uma TSE cujo objetivo é descrever como ocorre o controle da saúde dos pacientes com IC e os fatores que interferem neste controle, utilizando conceitos provenientes das linguagens padronizadas NANDA (*NANDA-International*), NIC (*Nursing Interventions Classification*) e NOC (*Nursing Outcomes Classification*) e da Teoria do Déficit do Autocuidado, de Orem^(9)^. A IC é doença crônica, progressiva, com sintomas e sinais que resultam de qualquer comprometimento estrutural ou funcional do enchimento ou ejeção do sangue pelos ventrículos^(10)^. Seu tratamento envolve acompanhamento multiprofissional, uso de polifarmácia e, frequentemente, o uso de dispositivos para controle de arritmias ou de assistência ventricular, o que demanda do indivíduo a necessidade de boa adesão à terapêutica, além do reconhecimento dos sintomas de descompensação da doença^(11)^. O conceito central da TSE mencionada foi “controle da saúde”, tendo-se em vista que, a despeito da importância de um bom controle da saúde pra redução das hospitalizações e melhora da qualidade de vida, a literatura mostra que tais indivíduos apresentam dificuldade em gerir adequadamente sua saúde^(9)^. Tal teoria foi construída entre 2019 e 2020 e – por entender que uma TSE deve apresentar a estrutura proposta pela literatura^(3)^, o que inclui ser facilmente aplicável à prática clínica – optou-se por submetê-la à avaliação por especialistas para avaliar sua adequação aos critérios de TSE descritos na literatura além de outros critérios referentes às teorias de enfermagem como um todo^(3,7)^.

Sendo assim, o objetivo deste estudo é descrever o processo de análise dos elementos intrínsecos da Teoria de Situação-específica para o controle da saúde na insuficiência cardíaca e apresentar sua respectiva atualização.

## Método

### Delineamento do estudo

Para a realização da análise da teoria construída, foi aplicada a técnica Delphi, uma ferramenta cujo objetivo consiste em se obter um consenso de especialistas, por uma série de questionários, acerca de um tema complexo^(12)^. A escolha do método deveu-se à escassez de metateoristas, no cenário brasileiro, que pudessem contribuir com a avaliação da Teoria e, conforme sugere a literatura, a obtenção de consenso de um pequeno grupo de especialistas pode ser útil para chegar a resultados consistentes, embora seu uso na validação de teorias de enfermagem ainda seja modesto^(5)^. De acordo com a literatura, não existe consenso sobre a quantidade mínima de indivíduos a serem escolhidos como juízes e nem os critérios que devem ser aplicados para sua escolha, devendo ficar a critério dos pesquisadores^(12)^. Estudos semelhantes utilizaram de três a sete juízes^(13-14)^, sendo que, para este estudo, os autores concordaram que seria necessário um mínimo de 12 experts, tendo em vista a perda de experts a cada rodada^(15)^. Para a apresentação das etapas do estudo, foi utilizado o *checklist* SQUIRE^(16)^.

### Critérios de seleção dos especialistas

O recrutamento dos juízes foi feito unindo dados provenientes de nossa experiência em pesquisa e por busca na Plataforma Lattes do portal do Conselho Nacional de Desenvolvimento Científico e Tecnológico (CNPq). Foram selecionados enfermeiros cuja expertise envolvia conhecimentos em Enfermagem em Cardiologia, em teorias de Enfermagem, em insuficiência cardíaca e/ou terminologias de Enfermagem. Para a seleção dos especialistas considerou-se não somente o conhecimento sobre a construção de teorias em si, mas também o conhecimento sobre a população estudada e sobre a estrutura que alicerçou a construção de seus conceitos (sistemas de linguagens padronizadas de enfermagem).

Os especialistas foram caracterizados de acordo com sua idade e sexo e, para sua caracterização, com relação à formação acadêmica e experiência clínica e no ensino, foram adotados os critérios estabelecidos por Benner, Tanner e Chesla^(17)^ em 2009 para a seleção e classificação dos juízes: experiência prática determinada pelo tempo de atuação em Cardiologia; e a experiência acadêmica determinada pela participação em grupos de pesquisas sobre: Cardiologia, linguagens padronizadas e/ou teorias de Enfermagem, bem como a produção de conhecimento e a publicação de artigos/trabalhos científicos nas temáticas referidas. Vale destacar que os intervalos construídos para os tempos de prática e de ensino foram estabelecidos pelos próprios autores a partir dos anos completos mínimo e máximo apresentados pelos juízes. O critério “qualificação” recebeu notas de 1 a 5 (1 – graduação; 2- residência; 3 – mestrado; 4 – doutorado; 5 - pós-doutorado). Para o critério “pesquisa para titulação” e a “publicação de artigos” nas temáticas sugeridas, atribuiu-se notas 0 para ausência e 1 para presença. O nível de expertise foi calculado pela média simples das pontuações obtidas, conforme apresentado na Figura 1.

**Figura 1 -:** Parâmetros para a classificação dos juízes quanto ao nível de expertise de Benner, Tanner e Chesla. São Paulo, Brasil, 2022

*W = Anos (intervalo construído a partir dos tempos mínimos e máximos de prática); ^†^X = Anos (intervalo construído a partir dos tempos mínimos e máximos de experiência no ensino); ^‡^Y = Participação em grupo de pesquisa sobre a temática; ^§^Z = Formação e produção científica sobre a temática; ^||^Z1 = Formação acadêmica; ^¶^Z2 = Dissertação ou tese sobre a temática; **Z3 = Artigos publicados sobre a temática

Estes parâmetros levaram à classificação dos juízes segundo o nível de expertise^(17)^, conforme descreve a Figura 2.

O convite, feito via *e-mail*, incluiu a descrição dos objetivos do estudo e o Termo de Consentimento Livre e Esclarecido (TCLE). Após o aceite e assinatura do TCLE, os juízes selecionados receberam a teoria na íntegra e preencheram o instrumento de análise. A coleta de dados ocorreu entra agosto de 2021 e abril de 2022.

**Figura 2 -:** Nível de expertise dos juízes. São Paulo, Brasil, 2022

### Elementos avaliados pelos especialistas

Os elementos constantes no instrumento de análise, compreendiam os aqueles sugeridos pela literatura^(3,7)^. Segundo a literatura, as teorias de situação-específica devem conter as seguintes propriedades^(3)^:

- *Baixo nível de abstração*: menos abstratas do que teorias de médio alcance e mais abstratas do que relatos de experiência, *guidelines*, protocolos, plano de cuidados e planos de ação;

- *Reflexão de um fenômeno específico de Enfermagem*: reflete um campo particular da prática; tem como objetivo desenvolver e compreender populações específicas e fornece estruturas ou projetos para ação;

- *Contexto*: devem abordar o contexto social, histórico e cultural; seu escopo é limitado a situações ou populações específicas;

- *Conexão prontamente acessível à pesquisa e prática de Enfermagem*: deve fornecer uma ligação entre teoria, pesquisa e prática;

- *Reflexão de diversidades no fenômeno de Enfermagem*: deve incluir diversidades, que abordam não somente aspectos culturais e políticos, mas também gênero, religiosidade, educação, dentre outros aspectos;

- *Generalização limitada*: por refletir diversidades e complexidades de um fenômeno de Enfermagem específico, a generalização é limitada;

Uma teoria ainda pode ser avaliada com relação à sua significância e adequação pragmática e os seus elementos no que tange à sua consistência interna, parcimônia, adequação empírica e adequação pragmática^(7)^:

- *Significância*: justifica a importância da teoria para a disciplina de Enfermagem;

- *Consistência interna*: avalia se os conceitos da teoria apresentam clareza e consistência semântica;

- *Parcimônia*: este critério exige que uma teoria seja declarada da maneira mais econômica possível, sem simplificar demais os fenômenos de interesse, ou seja, o melhor é que sejam necessários poucos conceitos e proposições para explicar completamente os fenômenos da teoria;

- *Adequação pragmática:* utilidade da teoria na prática. Ao revisar as descrições da teoria, será analisado se ela realmente pode ser utilizada no mundo real, na prática clínica, ou seja, é avaliada sua viabilidade.

A adequação empírica, também sugerida pelo referencial utilizado^(7)^, não foi avaliada pois analisa se as afirmações feitas pela teoria são congruentes com evidências empíricas encontradas nos estudos que a utilizam como base para a pesquisa, o que não era possível, já que se tratava de uma teoria recém-construída.

As propriedades das TSE e os critérios de significância e adequação pragmática foram aplicados à teoria como um todo; os conceitos foram avaliados de acordo com a consistência interna, as proposições e os pressupostos foram avaliados quanto à parcimônia.

A análise do pictograma foi realizada utilizando os seguintes critérios^(6)^:

-Representação gráfica e visual da teoria;

-Representação lógica;

-Clareza.

Cada item foi avaliado com relação à frequência e à proporção de concordância, sendo considerado como adequados os itens que obtiveram concordância igual ou superior a 80% entre os juízes^(18)^.

### 1ª rodada

Envio, via eletrônica: documento com descrição sucinta do método de construção teórica, a versão preliminar da teoria construída e um instrumento em que os juízes deveriam apontar seu grau de concordância com relação às propriedades da teoria, além de sua significância, consistência interna, parcimônia e adequação pragmática, e características específicas do pictograma. Após 15 dias, foi enviado um lembrete aos não respondentes e aguardou-se mais 15 dias. Devido à baixa resposta dos juízes, após o segundo lembrete, foi enviado novo *e-mail*, solicitando o preenchimento de resposta. Alguns juízes demonstraram interesse em participar após o segundo lembrete, porém não responderam dentro do prazo preestabelecido. Nesses casos, realizou-se contato telefônico e aguardou-se mais 20 dias. Embora, conforme mencionado, para a realização de Delphi, não exista um número mínimo de juízes, considerou-se um número mínimo de 12 especialistas como ideal para analisar este estudo. Dessa maneira, postergou-se o prazo de resposta, de modo a se obter a quantidade mínima desejada, fazendo com que o prazo total tenha sido de quatro meses. Após esse prazo, o processo de análise foi finalizado para a realização de coleta e análise dos dados da primeira rodada.

O instrumento enviado aos juízes foi composto de duas partes. Parte A: caracterização demográfica, acadêmica e profissional; parte B: questões sobre a análise da teoria propriamente dita. Tal instrumento propunha a análise da teoria como um todo, avaliando: as propriedades da teoria, a consistência interna dos conceitos, parcimônia e adequação pragmática, além de elementos específicos como: clareza, pertinência e relevância para o pictograma. Para cada item, os sujeitos deveriam informar o grau de concordância em uma escala de Likert de cinco pontos, variando de “discordo totalmente” a “concordo totalmente”, além de um espaço em aberto para sugestões de alterações e melhorias para cada item avaliado.

Os dados coletados na primeira rodada foram tabulados em planilhas do *Excel* e analisados por meio de estatística descritiva pelo *software* R. Cada item foi avaliado com relação à frequência e à proporção de concordância, sendo considerado como adequado o item que obteve concordância igual ou superior a 80% entre os juízes. Caso o juiz assinalasse “não concordo, nem discordo” (3 pontos), “discordo um pouco” (2 pontos) ou “discordo totalmente” (1 ponto), foi-lhe solicitado que oferecesse sugestões acerca das modificações necessárias na teoria. Para cada proporção foram calculados os intervalos de confiança de 95%, utilizando o método de Wald. O intervalo de confiança foi usado para estabelecer com 95% de confiança que a estimativa de proporção encontrada era estatisticamente igual ou superior ao ponto de corte estabelecido (80%).

### 2ª rodada

A segunda rodada ocorreu após a implementação das alterações sugeridas na primeira rodada e sua operacionalização foi de modo semelhante ao da anterior. Na segunda rodada, foram apresentados, aos especialistas, a teoria com suas modificações, o instrumento de análise e um feedback geral das alterações realizadas mediante as sugestões feitas na primeira rodada, as proporções de concordância encontradas para os elementos, e as sugestões propostas. Todo material foi enviado para os especialistas, via *e-mail*, com lembretes a cada 15 dias, sendo que, após três meses, com o objetivo de obter-se o maior número de experts participantes, foi realizado lembrete via contato telefônico com aqueles que não haviam respondido aos *e-mails* enviados. Esta etapa ocorreu entre dezembro de 2021 e abril de 2022.

### Aspectos éticos

O estudo foi aprovado pelo Comitê de Ética e Pesquisa (CEP) da Universidade Federal de São Paulo, sob parecer n. 3.903.496. Todos os preceitos éticos previstos na Resolução 466/2012^(19)^ foram atendidos. Foi enviada eletronicamente (via *e-mail*) uma carta convite aos especialistas selecionados juntamente com o Termo de Consentimento Livre e Esclarecido (TCLE). No TCLE foram apresentados os objetivos da pesquisa, seu aspecto voluntário e os procedimentos de coleta de dados. Foi considerado o aceite em participar da pesquisa os pesquisadores que devolveram o TCLE assinado.

## Resultados

Para a realização da análise da teoria, foram selecionados 22 enfermeiros com conhecimento em Enfermagem em Cardiologia, teorias de Enfermagem, insuficiência cardíaca e/ou terminologias de Enfermagem. Após o convite e lembretes enviados a cada 15 dias, no período de um mês, somente dois experts haviam respondido ao instrumento de análise. Dessa maneira, novos lembretes foram enviados, no período de quatro meses com intervalos de 15 dias, sendo possível obter resposta de um total de 15 experts para a primeira rodada de análise.

### Caracterização dos especialistas

Os especialistas selecionados para analisar a versão preliminar da teoria construída foram enfermeiros com amplo conhecimento e experiência em Cardiologia, linguagens padronizadas e/ou teorias de Enfermagem. O grupo foi composto por dois homens e 13 mulheres, com idade entre 31 e 62 anos. Para caracterizar os especialistas de acordo com sua formação acadêmica e experiência clínica e no ensino, foram adotados os critérios estabelecidos previamente^(11)^ e o nível de expertise foi calculado pela média simples das pontuações obtidas.

A partir dessa classificação, identificou-se que os juízes que realizaram a análise eram em sua maioria “competentes” (oito) ou “proficientes” (seis), sendo que somente um foi classificado como “perito”, o que denota um nível de expertise significativo dos juízes envolvidos na análise deste estudo.

### Resultados da 1ª rodada de análise

Após o preenchimento da primeira parte do instrumento de análise, os especialistas apontaram o seu grau de concordância com relação aos itens descritos. O instrumento completo continha 25 itens, sendo que os itens 1 a 19 abordavam a análise da teoria (teoria como um todo, conceitos, pressupostos e proposições) e os itens 20 a 25 estavam relacionados à análise do pictograma. Para a análise de concordância, foram compiladas as respostas “concordo” e “concordo totalmente”, bem como as respostas “discordo” e “discordo totalmente”. As respostas neutras (não concordo nem discordo) não foram consideradas para efeito de análise. Cada item foi avaliado com relação à frequência e à proporção de concordância, sendo considerado como adequados os itens que obtiveram concordância igual ou superior a 80% entre os juízes, conforme consta na Tabela 1.

**Tabela 1 -:** Resultados da avaliação dos juízes na primeira rodada de avaliação dos itens, conforme percentual de juízes que afirmaram concordar com os mesmos (n = 15*). São Paulo, Brasil, 2022

**Itens**	**n** ^†^	**%** ^‡^	**IC 95%** ^§^
Item 1- utilização de conceitos pouco abstratos para explicar o controle da saúde de pacientes com insuficiência cardíaca.	15	100,0	74,6 – 100,0
Item 2 - apresentação de forma explícita da situação específica abordada (controle da saúde de indivíduos com insuficiência cardíaca)	15	100,0	74,6 – 100,0
Item 3 – fornecimento de uma estrutura que representa especificamente o controle da saúde de pacientes com insuficiência cardíaca.	15	100,0	74,6 – 100,0
Item 4 - abordagem integrativa para a construção da teoria	12	80,0	51,4 – 94,7
Item 5 - especificidade da teoria à população ambulatorial com insuficiência cardíaca	13	86,7	58,4 – 97,6
Item 6- possibilidade de conexão das proposições entre a teoria e a prática	13	92,9	64,2 – 99,6
Item 7- fornece direções para sua utilização na pesquisa.	14	100,0	73,2 – 100,0
Item 8 - permite o estabelecimento de guias para a prática	14	100,0	73,2 – 100,0
Item 9 - incorporação de aspectos culturais, econômicos e clínicos do paciente com IC ^||^ .	13	86,7	58,4 – 97,6
Item 10 – incorporação das barreiras, dificuldades encontradas da população com insuficiência cardíaca para o controle de sua saúde	15	100,0	74,6 – 100,0
Item 11- preenchimento de lacuna de conhecimento existente.	14	100,0	73,2 – 100,0
Item 12 – ampliação de um conhecimento existente.	14	93,3	66,0 – 99,6
Item 13 – apresentação explícita do modelo conceitual de onde a teoria foi derivada	15	100,0	74,6 – 100,0
Item 14 - possibilidade de identificação das habilidades interpessoais e psicomotoras dos enfermeiros que são necessárias à sua aplicação.	15	100,0	74,6 – 100,0
Item 15 - viabilidade de implementação de protocolos clínicos derivados da teoria	15	100,0	74,6 – 100,0
Item 16 – possibilidade de atingir bons resultados com as ações de Enfermagem propostas pela teoria	14	100,0	73,2 – 100,0
Item17 - o fenômeno estudado (controle da saúde) é expresso adequadamente pelos conceitos	14	93,3	66,0 – 99,6
Item 18- possibilidade de categorizar e interpretar o fenômeno abrangido pela teoria por meio dos conceitos	13	86,7	58,4 – 97,6
Item 19 – abordagem do conteúdo de forma clara e concisa	15	100,0	74,6 – 100,0
Item 20 – representação gráfica e visual de forma adequada pelo pictograma	14	93,3	66,0 – 99,6
Item 21- possibilidade de compreender melhor os conceitos da teoria pelo pictograma	13	92,9	64,2 – 99,6
Item 22 – representação dos principais conceitos, no pictograma	14	93,3	66,0 – 99,6
Item 23 – relevância do pictograma para a teoria como um todo	15	100,0	74,6 – 100,0
Item 24 - apresentação lógica da relação entre os conceitos no pictograma	13	86,7	58,4 – 97,6
Item 25 – possibilidade de compreender melhor o texto e as relações entre os conceitos por meio do pictograma	15	100,0	74,6 – 100,0

*Total de experts; ^†^n = Total de experts que responderam estar de acordo com o item avaliado; ^‡^% = Proporção de experts que concordaram com o item avaliado; ^§^IC95% = Intervalo de confiança de 95%, ^||^IC = Insuficiência cardíaca. Nota: Itens com n<15 foram aqueles que não obtiveram resposta e/ou resposta “não concordo nem discordo”

Embora já, na primeira rodada, tenha-se obtido concordância superior a 80% em todos os itens analisados, alguns itens apresentaram um intervalo de confiança muito amplo e, por esse motivo, foram implementadas melhorias no corpo da teoria, norteadas pelas sugestões propostas pelos juízes. Um resumo das sugestões de melhorias que foram propostas para esses itens é apresentado na Figura 3.

**Figura 3 -:** Itens avaliados que apresentaram intervalo de confiança amplo, sugestões de melhorias na teoria e ações realizadas pela autora. São Paulo, Brasil, 2022

*IC = Insuficiência cardíaca; ^†^NIC = *Nursing Interventions Classification*

### Resultados da 2ª rodada de análise

Após terem sido realizadas as alterações sugeridas na primeira rodada, o texto atualizado da TSE construída foi enviado novamente aos mesmos especialistas da 1ª rodada, juntamente com um relatório informando os resultados da primeira rodada e as alterações realizadas no texto e no pictograma, de acordo com as sugestões recebidas.

A teoria revisada foi enviada na íntegra aos especialistas, com as alterações no texto evidenciadas na cor azul juntamente com um novo instrumento de análise, desta vez somente com os itens que foram revisados, para serem respondidos, totalizando oito itens. Os itens de 1 a 5 eram referentes à teoria e seus elementos, enquanto os itens de 6 a 8 eram relativos ao pictograma. O tempo desde o envio do primeiro e-mail, nesta segunda fase, com a solicitação de preenchimento da análise e as respostas do último especialista, foi de cerca de três meses, sendo que na 2ª rodada obteve-se o retorno de 14 especialistas, um a menos do que na 1ª rodada.

A partir do resultado desta segunda rodada, obteve-se concordância de 100% em praticamente todos os itens, bem como redução do intervalo de confiança. Os itens que apresentaram concordância inferior a 100% foram: item 2, referente à possibilidade de generalização da teoria (71,4%), e item 8 (92,9%), referente à clareza do pictograma.

A partir das sugestões que já haviam sido dadas na primeira rodada, as autoras assumem que, embora a teoria tenha sido desenvolvida para ser específica à população ambulatorial com IC, ela pode também ser aplicada na esfera de atenção primária à saúde, uma vez que os fatores condicionantes que afetam o controle da saúde do indivíduo atendido neste nível de atenção à saúde podem ser semelhantes, tornando-a útil também nesse cenário. Essa hipótese poderá ser confirmada ou refutada após a elaboração futura de indicadores que permitam a validação clínica da teoria. Com relação ao pictograma, como a concordância obtida foi superior a 80%, a versão enviada para análise foi tida como apropriada. Sendo assim, a versão analisada pelos juízes na segunda rodada de análise, por apresentarem altos níveis de concordância nos elementos avaliados, foi considerada como adequada para constituir-se na versão final da teoria.

## Discussão

A partir da análise dos especialistas, a TSE para o controle da saúde na IC pôde ter seus conceitos revistos e esclarecidos e suas interrelações apresentadas de modo mais claro no pictograma. Essa teoria incialmente foi construída com o objetivo de descrever o controle da saúde de pessoas com IC em atendimento ambulatorial, contudo, após a análise de especialistas, sugere-se que ela pode ser útil também no âmbito da atenção primária à saúde, por ser frequentemente o primeiro ponto de contato do usuário com o sistema de saúde e ser um ambiente que pode promover educação à saúde e melhorar a adesão ao tratamento.

Considera-se ainda que o enfermeiro pode educar e orientar o paciente nos mais diversos cenários de atendimento e embora o cenário ambulatorial, ou em nível de atenção primária, seja mais propício para que isso ocorra, compreende-se que a testagem futura da teoria em outros ambientes pode ser possível.

### A teoria revisada

Compreende-se que o indivíduo com IC que é capaz de controlar sua saúde utiliza seu livre arbítrio para desempenhar ações com a finalidade de manter sua vida, saúde e bem-estar. De acordo com a literatura^(20)^, esse processo envolve ações para a manutenção da estabilidade fisiológica, reconhecimento dos sintomas que evidenciam piora clínica e ações adequadas, quando os sintomas de piora são observados. Os elementos que influenciam o controle da saúde do indivíduo com IC são diversos e é importante que sejam de conhecimento da equipe multiprofissional, para que, assim, seja feito um planejamento apropriado das intervenções a serem implementadas. Por tal motivo, é importante que a saúde do indivíduo seja compreendida além do contexto biológico, compreendendo-se o contexto em que ele está inserido, como, por exemplo, sua constituição familiar, aspectos culturais e biopsicossociais, tal qual preconiza o princípio de Integralidade do Sistema Único de Saúde (SUS)^(21)^.

Para que o indivíduo seja capaz de controlar sua saúde, deve primeiramente desejar comprometer-se com a obtenção desse controle. Para que o controle da saúde seja alcançado, ele deve empenhar-se para desenvolver competências e habilidades específicas, que serão evidenciadas por meio de comportamentos de controle da saúde.

Os elementos que, em conjunto, podem atuar de forma favorável ou desfavorável no controle adequado do estado de saúde do indivíduo com IC são denominados: fatores condicionantes básicos. Algumas características tornam os indivíduos mais suscetíveis a não controlarem sua saúde de modo satisfatório, categorizando-os como “população em risco” de apresentar controle ineficaz da saúde. Já alguns elementos, como a presença de comorbidades, classe funcional elevada, dispositivos médicos relacionados à IC e tratamento medicamentoso complexo, são compreendidos como “condições associadas” podendo, também, levar o indivíduo a apresentar um controle ineficaz da saúde.

Em caso de um controle da saúde ineficiente, o indivíduo apresentará o DE Controle Ineficaz da Saúde [00078], evidenciando a necessidade de intervenções de Enfermagem. Em caso de intervenções eficientes, o esperado é que o controle da saúde seja atingido e, nessa hipótese, novas intervenções educativas e de reforço ao comportamento positivo deverão ser implementadas. Dessa forma, quanto mais elementos influenciarem negativamente o controle da saúde do indivíduo, maior será a demanda por intervenções de Enfermagem para auxiliá-lo a atingir este controle. As demandas individuais por intervenções que estimulem ou levem ao controle da saúde devem ser investigadas, de modo a identificar os pontos-chave a serem abordados pelo enfermeiro no desenvolvimento de seu plano terapêutico. As atividades de Enfermagem propostas compõem as intervenções da NIC Cuidados cardíacos [4040], Educação em saúde [5510] e Ensino: processo de doença [5602]. O enfermeiro deve atuar na orientação, monitoração e incentivo de comportamentos voltados ao controle da saúde, e, para tanto, pode utilizar como guia os indicadores dos resultados NOC Autocontrole da doença cardíaca [1617] e Comportamento de adesão [1600].

Na construção da primeira versão da teoria, após o estabelecimento dos conceitos teóricos e de suas inter-relações, foram estabelecidos seus pressupostos e proposições^(7)^. Tais componentes, após análise dos especialistas, permaneceram inalterados e são apresentados a seguir:

### Pressupostos

- Comprometer-se com o controle da saúde na IC requer capacidade para cuidar de si mesmo;

- A capacidade de controlar a saúde pode ser afetada por fatores que são inerentes ao indivíduo, como crenças e valores além daqueles relacionados à complexidade do sistema de saúde;

- O compromisso com o cuidado à saúde e os esforços em controlar a saúde na IC são influenciados por fatores intrínsecos e extrínsecos ao indivíduo;

- O compromisso com o cuidado à saúde e os esforços em controlar a saúde na IC habilitam o indivíduo em relação ao conhecimento sobre o que fazer e como agir, quando ocorrem sintomas de descompensação;

- Os enfermeiros promovem formas de auxiliar os indivíduos com IC, de acordo com as suas necessidades e dificuldades para controlarem sua saúde;

- A enfermagem é um dos serviços de saúde que é capaz de auxiliar os indivíduos com IC a controlarem sua saúde satisfatoriamente.

### Proposições

- Mudanças no controle da saúde de indivíduos com IC estão relacionadas a mudanças de comportamentos que podem ser expressos pelos indicadores dos resultados NOC Comportamento de adesão [1600] e Autocontrole da doença cardíaca [1617]^(22-24)^;

- Os indivíduos com IC com Controle Ineficaz da Saúde podem expressar as seguintes características: dificuldade com o regime prescrito, escolhas na vida diária ineficazes para atingir as metas de saúde, falha em agir para reduzir os fatores de risco, falha em incluir o regime de tratamento na vida diária^(25-26)^;

- O comprometimento com o controle da saúde do indivíduo com IC pode ser influenciado por fatores condicionantes, como: orientação sociocultural^(27)^, estado de saúde, fatores do sistema de saúde, sistemas de família^(25-26,28)^, padrões de vida, disponibilidade de recursos e adequação^(25,28-29)^, fatores ambientais, barreiras e benefícios percebidos, conflito de decisão, conhecimento insuficiente^(30)^, sentimento de impotência, suscetibilidade percebida^(31)^, demandas excessivas, dificuldade de controlar um regime de tratamento complexo^(32)^.

O pictograma que expressa a relação entre os conceitos foi atualizado conforme as sugestões propostas, demonstrando a interação entre os conceitos desta TSE (Figura 4).

**Figura 4 -:** Pictograma envolvendo metaparadigmas, conceitos centrais e periféricos da Teoria Situação-específica para o controle da saúde na insuficiência cardíaca. São Paulo, Brasil, 2022

A despeito da importância da boa gestão da saúde para a melhoria da qualidade de vida e redução das hospitalizações, é sabido que muitos pacientes com IC apresentam o DE Controle Ineficaz da Saúde. Diversos fatores internos e externos ao indivíduo influenciam a aquisição do controle da saúde e, embora nem todos sejam passíveis de intervenções de Enfermagem, sua identificação pode facilitar o processo diagnóstico por parte do enfermeiro.

Ao guiar o enfermeiro na identificação tanto dos aspectos clínicos que indicam que a doença não está sob controle, quanto de fatores ambientais e individuais que interferem na capacidade de o indivíduo controlar sua saúde adequadamente, esta teoria visa contribuir para que sejam adotadas estratégias que estimulem o indivíduo com IC a incluir o regime terapêutico em sua vida diária, melhorando sua qualidade de vida e sobrevida.

Suas proposições, refletem as interações entre os conceitos estabelecidos e são passíveis de testagem futura com a população em questão. Para tanto, poderão ser utilizadas as atividades de Enfermagem propostas nas intervenções da NIC Cuidados cardíacos [4040], Educação em saúde [5510] e Ensino: processo de doença [5602] e os indicadores dos resultados da NOC Autocontrole da doença cardíaca [1617] e Comportamento de adesão [1600].

## Conclusão

A validação realizada por meio da técnica Delphi possibilitou que a teoria fosse avaliada por especialistas experientes e pudesse ter seu texto e pictograma aprimorados com vistas a refletir mais fielmente o fenômeno estudado (controle da saúde) e facilitar sua incorporação na prática diária dos enfermeiros que atendem indivíduos com IC, seja ambulatorialmente, seja em nível de atenção primária à saúde. Ademais, a análise realizada permitiu atestar que a teoria construída apresenta as propriedades e características esperadas de uma TSE.

Validar uma teoria, seja a partir de uma perspectiva interna, seja a partir de sua testagem empírica, não é uma tarefa fácil e requer, além de tempo e atenção, grande expertise com relação ao método de construção teórica e sobre a temática abordada. A técnica Delphi, apesar de ainda ser pouco utilizada para essa finalidade, mostra-se útil por permitir a obtenção de consenso sem a necessidade de um grande número de especialistas, o que é particularmente vantajoso em temas com escassez de experts. Além da necessidade de encontrar profissionais com a expertise adequada para realizar a validação proposta, uma grande dificuldade foi o tempo de resposta dos juízes a cada rodada, o que impactou no tempo de conclusão do estudo.

Após a validação interna e atualização, a TSE para o controle da saúde na IC oferece melhor compreensão acerca dos fatores que podem influenciar o controle da saúde dos indivíduos com IC. Por ser uma teoria alicerçada na Teoria de Enfermagem do Déficit do Autocuidado de Orem, e utilizar conceitos provenientes das linguagens padronizadas NANDA, NIC e NOC, já amplamente utilizados na prática clínica, espera-se que ela possa contribuir para eliminar a dicotomia entre pensamento e ação, de modo que o enfermeiro da prática possa diagnosticar claramente os aspectos que envolvem o controle da saúde de seus pacientes e como deve ser a atuação da Enfermagem no atendimento a esses indivíduos. Almeja-se que, futuramente, sejam construídos instrumentos que permitam a avaliação do indivíduo, de modo mais concreto e, para tanto, poderão ser utilizados os indicadores dos resultados NOC “comportamento de adesão” e “autocontrole da doença cardíaca” como norteadores.

Considerando que o atendimento ao indivíduo com insuficiência cardíaca envolve uma abordagem multiprofissional e que alguns fatores que influenciam o controle da saúde não podem ser abordados exclusivamente pela enfermagem, acredita-se que essa teoria também possa ser útil para outras disciplinas.

# Translation: Actualización de la Teoría de Situación Específica para el control de la salud en la insuficiencia cardíaca: estudio Delphi*

**DOI:** 10.1590/1518-8345.7663.4553

**Authors:** Gisele Saraiva Bispo Hirano, Viviane Martins da Silva, Alba Lucia Bottura Leite de Barros

**Affiliations:** 1 Centro Universitário das Faculdades Metropolitanas Unidas, São Paulo, SP, Brasil; 2 Universidade Federal do Ceará, Departamento de Enfermagem, Fortaleza, CE, Brasil; 3 Universidade Federal de São Paulo, Escola Paulista de Enfermagem, São Paulo, SP, Brasil

**Keywords:** Teoría de Enfermería, Insuficiencia Cardíaca, Terminología Normalizada de Enfermería, Autocuidado, Técnica Delphi, Enfermería

## Abstract

describir el proceso de análisis de los elementos intrínsecos de la Teoría de Situación Específica para el control de la salud en la insuficiencia cardíaca y presentar su respectiva actualización.

análisis de los elementos intrínsecos de la teoría utilizando la técnica Delphi. Se seleccionaron enfermeros cuya experiencia abarcaba conocimientos en Enfermería en Cardiología, teorías de Enfermería, insuficiencia cardíaca y/o terminologías de Enfermería.

la teoría fue analizada por 15 especialistas en la primera ronda y 14 en la segunda ronda; todos los ítems evaluados obtuvieron una concordancia superior al 80% en cuanto a su adecuación, tras dos rondas de análisis. La teoría mantuvo su estructura original; sin embargo, se actualizaron la relación entre los factores que influyen en el control de la salud del individuo con insuficiencia cardíaca y el pictograma.

la teoría actualizada proporciona una mejor comprensión de los factores que pueden influir en el control de la salud de los individuos con insuficiencia cardíaca y, aunque el enfoque de la teoría es el ámbito ambulatorio, se considera la posibilidad de su aplicación en otros escenarios. La técnica Delphi resultó útil para la validación teórica, considerando la especificidad del tema; no obstante, el tiempo de respuesta de los especialistas puede ser prolongado, lo que tiende a impactar en el tiempo de finalización del estudio.

## Destacados

**(1)** Utilización de Delphi para la validación interna de la Teoría de Situación Específica. **(2)** Descripción de las características de una Teoría de Situación Específica en enfermería. **(3)** Innovación con el uso de lenguajes estandarizados en la construcción de teoría. **(4)** Avances en el conocimiento sobre el control de la salud en la insuficiencia cardíaca. **(5)** Mejoramiento de la asistencia de enfermería a personas con insuficiencia cardíaca.

## Introdução

A lo largo del último siglo, la enfermería ha evolucionado de una ocupación a una profesión, lo que ha generado un creciente interés en construir un cuerpo de conocimiento propio, dejando atrás el empirismo para fundamentar la práctica clínica en evidencias científicas. Con la cientificación de la profesión, se comenzaron a desarrollar teorías de enfermería con el propósito de comprender mejor los fenómenos y las respuestas humanas que son de interés para la enfermería^(1-2)^. Las teorías de enfermería consisten en un conjunto de conceptos y proposiciones que ayudan a explicar, predecir y prescribir cuidados de enfermería, es decir, sustentan los saberes y la práctica de la enfermería, siendo, por lo tanto, fundamentales para el desarrollo de la profesión^(1)^.

Las teorías de enfermería pueden clasificarse, según su nivel de abstracción, en grandes teorías (más abstractas), teorías de alcance medio (nivel intermedio de abstracción) y teorías de situación específica (menos abstractas) o teorías prácticas, dependiendo de la literatura consultada. El término “teorías de situación específica” (TSE) fue utilizado por primera vez en 1999 por Im y Meleis, al describir teorías menos abstractas que las teorías de alcance medio y dirigidas a poblaciones y situaciones específicas que pudieran ser más fácilmente aplicables en la práctica clínica del enfermero^(3)^. Las propiedades de una TSE, descritas por Im y Meleis, son: bajo nivel de abstracción; reflexión sobre un fenómeno específico de enfermería, contexto, conexión prontamente accesible a la investigación y práctica de enfermería, reflejo de diversidades en el fenómeno de enfermería y generalización limitada^(3)^.

Para determinar la relevancia y calidad de una teoría, se sugiere que sea sometida a una evaluación de sus elementos intrínsecos o a una validación clínica a partir de pruebas empíricas^(4)^. La evaluación de sus elementos debe determinar si el enfoque epistemológico está en consonancia con lo esperado, según la referencia utilizada. Debido a la especificidad del tema, someter una teoría de enfermería a una evaluación de sus elementos intrínsecos puede ser una tarea ardua, ya que es necesario que los evaluadores posean una alta experiencia en este campo. De esta manera, se hace necesaria la elección de métodos de evaluación que sean no solo confiables, sino también viables desde el punto de vista metodológico.

La evaluación de una teoría, también denominada validación, es el principal método para verificar su calidad y adecuación a criterios preestablecidos, además de señalar necesidades de mejora para facilitar su aplicación. Dicha validación puede ser desde una perspectiva interna, es decir, aquella que verifica la adecuación de sus elementos intrínsecos, o externa, a través de pruebas empíricas en las que pueden utilizarse diversos autores como guía^(5)^, así como diferentes estructuras pueden emplearse para este tipo de análisis^(6-7)^. En el caso de las teorías de situación específica, puede utilizarse como guía la estructura que describe con mayor detalle las características de este tipo particular de teoría^(3,8)^.

En 2023, se publicó la TSE para el control de la salud en la insuficiencia cardíaca (IC), una TSE cuyo objetivo es describir cómo ocurre el control de la salud de los pacientes con IC y los factores que influyen en este control, utilizando conceptos provenientes de los lenguajes estandarizados NANDA (*NANDA*-*International*), NIC (*Nursing Interventions Classification*) y NOC (*Nursing Outcomes Classification*), además de la Teoría del Déficit del Autocuidado de Orem^(9)^. La IC es una enfermedad crónica y progresiva, cuyos síntomas y signos resultan de cualquier compromiso estructural o funcional del llenado o eyección de la sangre por los ventrículos^(10)^. Su tratamiento implica un seguimiento multiprofesional, el uso de polifarmacia y, con frecuencia, el uso de dispositivos para el control de arritmias o asistencia ventricular, lo que exige del individuo una buena adherencia a la terapia, además del reconocimiento de los síntomas de descompensación de la enfermedad^(11)^. El concepto central de la mencionada TSE fue el “control de la salud”, considerando que, a pesar de la importancia de un buen control de la salud para la reducción de hospitalizaciones y la mejora de la calidad de vida, la literatura muestra que estos individuos presentan dificultades para gestionar adecuadamente su salud^(9)^. Dicha teoría fue construida entre 2019 y 2020 y, al considerar que una TSE debe presentar la estructura propuesta en la literatura^(3)^, lo que incluye su fácil aplicación en la práctica clínica, se optó por someterla a la evaluación de especialistas para verificar su adecuación a los criterios de TSE descritos en la literatura, además de otros criterios referentes a las teorías de enfermería en general^(3,7)^.

Por lo tanto, el objetivo de este estudio es describir el proceso de análisis de los elementos intrínsecos de la TSE para el control de la salud en la insuficiencia cardíaca y presentar su respectiva actualización.

## Método

### Diseño del estudio

Para la realización del análisis de la teoría construida, se aplicó la técnica Delphi, una herramienta cuyo objetivo es obtener consenso entre expertos, mediante una serie de cuestionarios, sobre un tema complejo^(12)^. La elección del método se debió a la escasez de metateoristas en el contexto brasileño que pudieran contribuir con la evaluación de la teoría y, según sugiere la literatura, la obtención de consenso de un pequeño grupo de expertos puede ser útil para alcanzar resultados consistentes, aunque su uso en la validación de teorías de enfermería aún sea limitado^(5)^. De acuerdo con la literatura, no existe un consenso sobre la cantidad mínima de individuos a ser seleccionados como jueces ni sobre los criterios que deben aplicarse para su elección, quedando a criterio de los investigadores^(12)^. Estudios similares han utilizado de tres a siete jueces^(13-14)^, y para este estudio, los autores acordaron que sería necesario un mínimo de 12 expertos, considerando la posible pérdida de participantes en cada ronda^(15)^. Para la presentación de las etapas del estudio, se utilizó la lista de verificación SQUIRE^(16)^.

### Criterios de selección de los expertos

El reclutamiento de los jueces se realizó combinando datos provenientes de nuestra experiencia en investigación y mediante la búsqueda en la Plataforma Lattes del portal del *Conselho Nacional de Desenvolvimento Científico e Tecnológico* (CNPq). Se seleccionaron enfermeros cuya experiencia abarcaba conocimientos en Enfermería en Cardiología, teorías de Enfermería, insuficiencia cardíaca y/o terminologías de Enfermería. Para la selección de los expertos, se consideró no solo el conocimiento sobre la construcción de teorías en sí, sino también el conocimiento sobre la población estudiada y sobre la estructura que sustentó la construcción de sus conceptos (sistemas de lenguajes estandarizados de enfermería).

Los expertos fueron caracterizados según su edad y sexo y, para su caracterización en cuanto a formación académica y experiencia clínica y docente, se adoptaron los criterios establecidos por Benner, Tanner y Chesla^(17)^ en 2009 para la selección y clasificación de los jueces: la experiencia práctica se determinó por el tiempo de actuación en Cardiología; y la experiencia académica, por la participación en grupos de investigación sobre Cardiología, lenguajes estandarizados y/o teorías de Enfermería, así como por la producción de conocimiento y la publicación de artículos o trabajos científicos en las temáticas mencionadas. Cabe destacar que los intervalos construidos para los tiempos de práctica y enseñanza fueron establecidos por los propios autores a partir de los años completos mínimo y máximo presentados por los jueces. El criterio de “cualificación” recibió puntuaciones de 1 a 5 (1 – grado; 2 – residencia; 3 – maestría; 4 – doctorado; 5 – posdoctorado). Para el criterio “investigación para titulación” y “publicación de artículos” en las temáticas sugeridas, se asignó una puntuación de 0 para ausencia y 1 para presencia. El nivel de expertise se calculó mediante el promedio simple de las puntuaciones obtenidas, según se presenta en la Figura 1.

**Figura 1 -:** Parámetros para la clasificación de los jueces según el nivel de expertise de Benner, Tanner y Chesla. São Paulo, Brasil, 2022

*W = Años (intervalo construido a partir de los tiempos mínimos y máximos de práctica); ^†^X = Años (intervalo construido a partir de los tiempos mínimos y máximos de experiencia en enseñanza); ^‡^Y = Participación en grupos de investigación sobre la temática; ^§^Z = Formación y producción científica sobre la temática; ^||^Z1 = Formación académica; ^¶^Z2 = Disertación o tesis sobre la temática; **Z3 = Artículos publicados sobre la temática

Estos parámetros llevaron a la clasificación de los jueces según el nivel de expertise^(17)^, como se describe en la Figura 2.

La invitación, enviada por correo electrónico, incluyó la descripción de los objetivos del estudio y el Consentimiento Informado (CI). Tras la aceptación y firma del CI, los jueces seleccionados recibieron la teoría en su totalidad y completaron el instrumento de análisis. La recolección de datos se realizó entre agosto de 2021 y abril de 2022.

**Figura 2 -:** Nivel de expertise de los jueces. São Paulo, Brasil, 2022

### Elementos evaluados por los expertos

Los elementos incluidos en el instrumento de análisis correspondían a aquellos sugeridos por la literatura^(3,7)^.

Según la literatura, las teorías de situación específica deben contener las siguientes propiedades^(3)^:

- *Bajo nivel de abstracción:* menos abstractas que las teorías de alcance medio y más abstractas que los relatos de experiencia, *guidelines*, protocolos, planes de cuidados y planes de acción;

- *Reflexión de un fenómeno específico de Enfermería:* refleja un campo particular de la práctica; tiene como objetivo desarrollar y comprender poblaciones específicas y proporciona estructuras o diseños para la acción;

- *Contexto*: debe abordar el contexto social, histórico y cultural; su alcance está limitado a situaciones o poblaciones específicas;

- *Conexión prontamente accesible a la investigación y práctica de Enfermería:* debe proporcionar un vínculo entre teoría, investigación y práctica;

- *Reflexión de diversidades en el fenómeno de Enfermería:* debe incluir diversidades que aborden no solo aspectos culturales y políticos, sino también género, religiosidad, educación, entre otros;

- *Generalización limitada:* al reflejar diversidades y complejidades de un fenómeno específico de Enfermería, la generalización es limitada;

Una teoría también puede ser evaluada en cuanto a su significancia y adecuación pragmática, así como en relación con sus elementos de consistencia interna, parsimonia, adecuación empírica y adecuación pragmática^(7)^:

- *Significancia*: justifica la importancia de la teoría para la disciplina de Enfermería;

- *Consistencia interna*: evalúa si los conceptos de la teoría presentan claridad y consistencia semántica;

- *Parsimonia*: este criterio exige que una teoría sea expresada de la manera más concisa posible, sin simplificar excesivamente los fenómenos de interés. Es decir, se considera ideal que sean necesarios pocos conceptos y proposiciones para explicar completamente los fenómenos de la teoría;

- *Adecuación pragmática:* analiza la utilidad de la teoría en la práctica. Al revisar sus descripciones, se evalúa si realmente puede aplicarse en el mundo real, en la práctica clínica, es decir, su viabilidad.

La adecuación empírica, también sugerida por el marco de referencia utilizado^(7)^, no fue evaluada, ya que analiza si las afirmaciones hechas por la teoría son congruentes con las evidencias empíricas encontradas en estudios que la utilizan como base para la investigación. Esto no era posible en este caso, ya que se trataba de una teoría recién construida.

Las propiedades de las TSE y los criterios de significancia y adecuación pragmática fueron aplicados a la teoría en su conjunto. Los conceptos fueron evaluados en relación con la consistencia interna, mientras que las proposiciones y supuestos fueron evaluados en términos de parsimonia.

El análisis del pictograma se realizó utilizando los siguientes criterios^(6)^:

- Representación gráfica y visual de la teoría;

- Representación lógica;

- Claridad.

Cada elemento fue evaluado en términos de frecuencia y proporción de concordancia, considerándose adecuados aquellos ítems que obtuvieron una concordancia igual o superior al 80% entre los jueces^(18)^.

### Primera ronda

Envío electrónico: se remitió un documento con una descripción concisa del método de construcción teórica, la versión preliminar de la teoría y un instrumento en el que los jueces debían indicar su grado de acuerdo con respecto a las propiedades de la teoría, además de su significancia, consistencia interna, parsimonia, adecuación pragmática y características específicas del pictograma. Después de 15 días, se envió un recordatorio a los participantes que aún no habían respondido y se esperó otros 15 días. Debido a la baja tasa de respuesta, tras el segundo recordatorio, se envió un nuevo correo electrónico solicitando la cumplimentación del cuestionario. Algunos jueces manifestaron interés en participar después del segundo recordatorio, pero no enviaron sus respuestas dentro del plazo establecido. En estos casos, se realizó un contacto telefónico y se concedió un plazo adicional de 20 días. Aunque, como se mencionó anteriormente, para la técnica Delphi no existe un número mínimo de jueces, se consideró ideal contar con al menos 12 especialistas para analizar este estudio. Por lo tanto, se extendió el plazo de respuesta con el objetivo de alcanzar la cantidad mínima deseada, lo que llevó a que el tiempo total de recolección de respuestas fuera de cuatro meses. Finalizado este período, se dio por concluido el proceso de análisis para proceder a la recopilación y análisis de los datos de la primera ronda.

El instrumento enviado a los jueces estuvo compuesto por dos partes. Parte A: caracterización demográfica, académica y profesional; parte B: cuestiones sobre el análisis de la teoría propiamente dicha. Dicho instrumento proponía el análisis de la teoría en su conjunto, evaluando: las propiedades de la teoría, la consistencia interna de los conceptos, la parsimonia y la adecuación pragmática, además de elementos específicos como: claridad, pertinencia y relevancia del pictograma. Para cada ítem, los sujetos debían indicar el grado de concordancia en una escala de Likert de cinco puntos, que variaba de “totalmente en desacuerdo” a “totalmente de acuerdo”, además de un espacio abierto para sugerencias de modificaciones y mejoras para cada ítem evaluado.

Los datos recopilados en la primera ronda fueron tabulados en hojas de cálculo de Excel y analizados mediante estadística descriptiva con el *software* R. Cada ítem fue evaluado en función de la frecuencia y proporción de concordancia, considerándose adecuado aquel que obtuvo una concordancia igual o superior al 80% entre los jueces. En caso de que el juez marcara “ni de acuerdo ni en desacuerdo” (3 puntos), “en desacuerdo en cierta medida” (2 puntos) o “totalmente en desacuerdo” (1 punto), se le solicitó que ofreciera sugerencias sobre las modificaciones necesarias en la teoría. Para cada proporción se calcularon intervalos de confianza del 95% utilizando el método de Wald. El intervalo de confianza se empleó para establecer con un 95% de certeza que la estimación de proporción encontrada era estadísticamente igual o superior al punto de corte establecido (80%).

### Segunda ronda

La segunda ronda tuvo lugar después de la implementación de las modificaciones sugeridas en la primera ronda y su operacionalización se llevó a cabo de manera similar a la anterior. En esta segunda ronda, se presentó a los especialistas la teoría con sus modificaciones, el instrumento de análisis y un *feedback* general sobre los cambios realizados a partir de las sugerencias de la primera ronda, incluyendo las proporciones de concordancia obtenidas para los elementos y las sugerencias propuestas. Todo el material fue enviado a los especialistas por correo electrónico, con recordatorios cada 15 días. Después de tres meses, con el objetivo de obtener el mayor número posible de expertos participantes, se realizó un recordatorio mediante contacto telefónico con aquellos que no habían respondido a los correos electrónicos enviados. Esta etapa se llevó a cabo entre diciembre de 2021 y abril de 2022.

### Aspectos éticos

El estudio fue aprobado por el Comité de Ética en Investigación (CEI) de la *Universidade Federal de São Paulo*, bajo el dictamen n. 3.903.496. Se cumplieron todos los preceptos éticos establecidos en la Resolución 466/2012^(19)^. Se envió electrónicamente (por correo electrónico) una carta de invitación a los especialistas seleccionados, junto con el Consentimiento Informado (CI). En el CI se presentaron los objetivos de la investigación, su carácter voluntario y los procedimientos de recolección de datos. Se consideró como aceptación de participación en la investigación el envío del CI firmado por los investigadores.

## Resultados

Para la realización del análisis de la teoría, se seleccionaron 22 enfermeros con conocimientos en Enfermería en Cardiología, teorías de Enfermería, insuficiencia cardíaca y/o terminologías de Enfermería. Tras la invitación y los recordatorios enviados cada 15 días durante un mes, solo dos expertos habían respondido al instrumento de análisis. Por lo tanto, se enviaron nuevos recordatorios durante un período de cuatro meses, con intervalos de 15 días, logrando obtener la respuesta de un total de 15 expertos en la primera ronda de análisis.

### Caracterización de los expertos

Los expertos seleccionados para analizar la versión preliminar de la teoría construida fueron enfermeros con amplio conocimiento y experiencia en Cardiología, lenguajes estandarizados y/o teorías de Enfermería. El grupo estuvo compuesto por dos hombres y 13 mujeres, con edades entre 31 y 62 años. Para caracterizar a los especialistas de acuerdo con su formación académica y experiencia clínica y docente, se adoptaron los criterios previamente establecidos^(11)^ y el nivel de expertise se calculó mediante el promedio simple de las puntuaciones obtenidas.

A partir de esta clasificación, se identificó que la mayoría de los jueces que participaron en el análisis fueron clasificados como “competentes” (ocho) o “proficientes” (seis), mientras que solo uno fue clasificado como “experto”, lo que indica un nivel de expertise significativo entre los jueces involucrados en el análisis de este estudio.

### Resultados de la primera ronda de análisis

Tras completar la primera parte del instrumento de análisis, los especialistas indicaron su grado de concordancia con respecto a los ítems descritos. El instrumento completo contenía 25 ítems, de los cuales los ítems 1 a 19 abordaban el análisis de la teoría (teoría en su conjunto, conceptos, supuestos y proposiciones) y los ítems 20 a 25 estaban relacionados con el análisis del pictograma. Para el análisis de concordancia, se agruparon las respuestas “de acuerdo” y “totalmente de acuerdo”, así como las respuestas “en desacuerdo” y “totalmente en desacuerdo”. Las respuestas neutras (“ni de acuerdo ni en desacuerdo”) no fueron consideradas para efectos de análisis. Cada ítem fue evaluado en función de la frecuencia y la proporción de concordancia, considerándose adecuados aquellos que obtuvieron una concordancia igual o superior al 80% entre los jueces, conforme se presenta en la Tabla 1.

**Tabla 1 -:** Resultados de la evaluación de los jueces en la primera ronda de análisis de los ítems, según el porcentaje de jueces que afirmaron estar de acuerdo con los mismos (n = 15*). São Paulo, Brasil, 2022

Ítems	**n** ^†^	**%** ^‡^	**IC95%** ^§^
Ítem 1 - utilización de conceptos poco abstractos para explicar el control de la salud de pacientes con insuficiencia cardíaca	15	100,0	74,6 – 100,0
Ítem 2 - presentación explícita de la situación específica abordada (control de la salud de individuos con insuficiencia cardíaca)	15	100,0	74,6 – 100,0
Ítem 3 - suministro de una estructura que representa específicamente el control de la salud de pacientes con insuficiencia cardíaca	15	100,0	74,6 – 100,0
Ítem 4 - enfoque integrador para la construcción de la teoría	12	80,0	51,4 – 94,7
Ítem 5 - especificidad de la teoría para la población ambulatoria con insuficiencia cardíaca	13	86,7	58,4 – 97,6
Ítem 6 - posibilidad de conexión de las proposiciones entre la teoría y la práctica	13	92,9	64,2 – 99,6
Ítem 7 - proporciona directrices para su uso en la investigación	14	100,0	73,2 – 100,0
Ítem 8 - permite el establecimiento de guías para la práctica	14	100,0	73,2 – 100,0
Ítem 9 - incorporación de aspectos culturales, económicos y clínicos del paciente con IC ^||^ .	13	86,7	58,4 – 97,6
Ítem 10 - incorporación de barreras y dificultades encontradas por la población con insuficiencia cardíaca para el control de su salud	15	100,0	74,6 – 100,0
Ítem 11 - cobertura de una brecha de conocimiento existente	14	100,0	73,2 – 100,0
Ítem 12 - ampliación de un conocimiento existente	14	93,3	66,0 – 99,6
Ítem 13 - presentación explícita del modelo conceptual del cual se derivó la teoría	15	100,0	74,6 – 100,0
Ítem 14 - posibilidad de identificar las habilidades interpersonales y psicomotoras necesarias para su aplicación	15	100,0	74,6 – 100,0
Ítem 15 - viabilidad de implementación de protocolos clínicos derivados de la teoría	15	100,0	74,6 – 100,0
Ítem 16 - posibilidad de alcanzar buenos resultados con las acciones de Enfermería propuestas por la teoría	14	100,0	73,2 – 100,0
Ítem 17 - el fenómeno estudiado (control de la salud) está adecuadamente expresado a través de los conceptos	14	93,3	66,0 – 99,6
Ítem 18 - posibilidad de categorizar e interpretar el fenómeno abordado por la teoría a través de los conceptos	13	86,7	58,4 – 97,6
Ítem 19 - abordaje del contenido de manera clara y concisa	15	100,0	74,6 – 100,0
Ítem 20 - representación gráfica y visual adecuada mediante el pictograma	14	93,3	66,0 – 99,6
Ítem 21 - posibilidad de comprender mejor los conceptos de la teoría a través del pictograma	13	92,9	64,2 – 99,6
Ítem 22 - representación de los principales conceptos en el pictograma	14	93,3	66,0 – 99,6
Ítem 23 - relevancia del pictograma para la teoría en su conjunto	15	100,0	74,6 – 100,0
Ítem 24 - presentación lógica de la relación entre los conceptos en el pictograma	13	86,7	58,4 – 97,6
Ítem 25 - posibilidad de comprender mejor el texto y las relaciones entre los conceptos a través del pictograma	15	100,0	74,6 – 100,0

*n=15 = Total de expertos; ^†^n = Total de expertos que respondieron estar de acuerdo con el ítem evaluado; ^‡^% = Proporción de expertos que concordaron con el ítem evaluado; ^§^IC95% = Intervalo de confianza del 95%, ^||^IC = Insuficiencia cardíaca. Nota: Ítems con n<15 corresponden a aquellos que no obtuvieron respuesta y/o en los que la respuesta fue “ni de acuerdo ni en desacuerdo”

Aunque en la primera ronda se obtuvo un nivel de concordancia superior al 80% en todos los ítems analizados, algunos presentaron un intervalo de confianza muy amplio. Por esta razón, se implementaron mejoras en el cuerpo de la teoría, guiadas por las sugerencias propuestas por los jueces. Un resumen de las sugerencias de mejora propuestas para estos ítems se presenta en la Figura 3.

**Figura 3 -:** Ítems evaluados que presentaron un intervalo de confianza amplio, sugerencias de mejoras en la teoría y acciones realizadas por la autora. São Paulo, Brasil, 2022

*IC = Insuficiencia cardíaca; ^†^NIC = *Nursing Interventions Classification*

### Resultados de la segunda ronda de análisis

Tras la implementación de las modificaciones sugeridas en la primera ronda, el texto actualizado de la TSE construida fue enviado nuevamente a los mismos especialistas de la primera ronda, junto con un informe que detallaba los resultados obtenidos en la primera evaluación y las modificaciones realizadas en el texto y el pictograma, de acuerdo con las sugerencias recibidas.

La teoría revisada fue enviada en su totalidad a los especialistas, con las modificaciones en el texto resaltadas en color azul, junto con un nuevo instrumento de análisis que contenía únicamente los ítems que fueron revisados, sumando un total de ocho ítems. Los ítems del 1 al 5 estaban relacionados con la teoría y sus elementos, mientras que los ítems del 6 al 8 correspondían al pictograma. El período desde el envío del primer correo electrónico en esta segunda fase, con la solicitud de respuesta al instrumento de análisis, hasta la recepción de la última respuesta de los especialistas fue de aproximadamente tres meses. En esta segunda ronda, se obtuvo la respuesta de 14 especialistas, uno menos que en la primera ronda.

A partir de los resultados de esta segunda ronda, se logró una concordancia del 100 % en prácticamente todos los ítems, además de una reducción en el intervalo de confianza. Los ítems que presentaron una concordancia inferior al 100% fueron: ítem 2, referente a la posibilidad de generalización de la teoría (71,4%), y ítem 8, referente a la claridad del pictograma (92,9%).

A partir de las sugerencias ya formuladas en la primera ronda, las autoras asumen que, aunque la teoría fue desarrollada para ser específica a la población ambulatoria con IC, también puede aplicarse en la atención primaria de salud, dado que los factores condicionantes que afectan el control de la salud del individuo atendido en este nivel de atención pueden ser similares, lo que la hace potencialmente útil en este contexto. Esta hipótesis podrá ser confirmada o refutada en el futuro mediante la elaboración de indicadores que permitan la validación clínica de la teoría. En relación con el pictograma, dado que la concordancia obtenida fue superior al 80 %, la versión enviada para análisis fue considerada apropiada. Así, la versión analizada por los jueces en la segunda ronda, al presentar altos niveles de concordancia en los elementos evaluados, fue considerada adecuada para constituirse en la versión final de la teoría.

## Discusión

A partir del análisis de los especialistas, la TSE para el control de la salud en la IC tuvo sus conceptos revisados y aclarados, y sus interrelaciones fueron presentadas de manera más clara en el pictograma. Esta teoría fue inicialmente construida con el objetivo de describir el control de la salud de personas con IC en atención ambulatoria; sin embargo, tras el análisis de los especialistas, se sugiere que también podría ser útil en el ámbito de la atención primaria de salud, dado que este nivel de atención suele ser el primer punto de contacto del usuario con el sistema de salud y representa un entorno propicio para la promoción de la educación en salud y la mejora de la adherencia al tratamiento.

Se considera, además, que el enfermero puede educar y orientar al paciente en diversos escenarios de atención. Aunque el contexto ambulatorio o el nivel de atención primaria sean los más adecuados para este propósito, se entiende que la futura validación de la teoría en otros entornos podría ser factible.

### La teoría revisada

Se entiende que el individuo con IC que es capaz de controlar su salud utiliza su libre albedrío para llevar a cabo acciones con el propósito de mantener su vida, salud y bienestar. De acuerdo con la literatura^(20)^, este proceso implica acciones para el mantenimiento de la estabilidad fisiológica, reconocimiento de los síntomas que evidencian un deterioro clínico y la adopción de medidas adecuadas cuando estos síntomas son observados. Los elementos que influyen en el control de la salud del individuo con IC son diversos y deben ser conocidos por el equipo multiprofesional para que se realice una planificación adecuada de las intervenciones a implementar. Por esta razón, es fundamental comprender la salud del individuo más allá del contexto biológico, considerando el entorno en el que está inserto, como su constitución familiar, aspectos culturales y biopsicosociales, en línea con el principio de Integralidad del Sistema Único de Salud (SUS)^(21)^.

Para que el individuo sea capaz de controlar su salud, primero debe desear comprometerse con la obtención de dicho control. Para lograrlo, debe esforzarse en desarrollar competencias y habilidades específicas, que se evidenciarán a través de comportamientos de control de la salud.

Los elementos que, en conjunto, pueden influir de forma favorable o desfavorable en el control adecuado del estado de salud del individuo con IC son denominados factores condicionantes básicos. Algunas características hacen que ciertos individuos sean más susceptibles a no controlar su salud de manera satisfactoria, lo que los categoriza como población en riesgo de presentar un control ineficaz de la salud. Otros elementos, como la presencia de comorbilidades, clase funcional elevada, dispositivos médicos relacionados con la IC y un tratamiento farmacológico complejo, son considerados condiciones asociadas, que también pueden llevar al individuo a presentar un control ineficaz de la salud.

En caso de un control de la salud ineficiente, el individuo presentará el diagnóstico de enfermería Control Ineficaz de la Salud [00078], lo que evidencia la necesidad de intervenciones de Enfermería. Si las intervenciones son eficaces, se espera que el control de la salud sea alcanzado y, en esta hipótesis, deberán implementarse nuevas intervenciones educativas y de refuerzo del comportamiento positivo. De este modo, cuantos más elementos influyan negativamente en el control de la salud del individuo, mayor será la demanda de intervenciones de Enfermería para ayudarlo a lograr este control. Las demandas individuales de intervenciones que estimulen o faciliten el control de la salud deben ser investigadas para identificar los puntos clave que el enfermero debe abordar en el desarrollo de su plan terapéutico. Las actividades de Enfermería propuestas incluyen las intervenciones de la NIC Cuidados cardíacos [4040], Educación en salud [5510] y Enseñanza: proceso de enfermedad [5602]. El enfermero debe actuar en la orientación, monitoreo e incentivo de comportamientos dirigidos al control de la salud y, para ello, puede utilizar como guía los indicadores de los resultados NOC Autocontrol de la enfermedad cardíaca [1617] y Comportamiento de adherencia [1600].

En la construcción de la primera versión de la teoría, tras el establecimiento de los conceptos teóricos y sus interrelaciones, se definieron sus supuestos y proposiciones^(7)^. Tras el análisis de los especialistas, estos componentes permanecieron inalterados y se presentan a continuación:

### Supuestos

- Comprometerse con el control de la salud en la IC requiere la capacidad de autocuidado;

- La capacidad de controlar la salud puede verse afectada por factores inherentes al individuo, como creencias y valores, además de aquellos relacionados con la complejidad del sistema de salud;

- El compromiso con el cuidado de la salud y los esfuerzos por controlar la salud en la IC están influenciados por factores intrínsecos y extrínsecos al individuo;

- El compromiso con el cuidado de la salud y los esfuerzos por controlar la salud en la IC capacitan al individuo en cuanto al conocimiento sobre qué hacer y cómo actuar cuando aparecen síntomas de descompensación;

- Los enfermeros promueven formas de ayudar a los individuos con IC, de acuerdo con sus necesidades y dificultades para controlar su salud;

- La Enfermería es uno de los servicios de salud que puede ayudar a los individuos con IC a controlar su salud de manera satisfactoria.

### Proposiciones

- Los cambios en el control de la salud de individuos con IC están relacionados con cambios en los comportamientos, los cuales pueden ser expresados por los indicadores de los resultados NOC Comportamiento de adherencia [1600] y Autocontrol de la enfermedad cardíaca [1617]^(22-24)^;

- Los individuos con IC con Control Ineficaz de la Salud pueden expresar las siguientes características: dificultad con el régimen prescrito, elecciones ineficaces en la vida diaria para alcanzar las metas de salud, falta de acción para reducir los factores de riesgo, falta de integración del régimen de tratamiento en la vida diaria^(25-26)^;

- El compromiso con el control de la salud del individuo con IC puede estar influenciado por factores condicionantes como: orientación sociocultural^(27)^, estado de salud, factores del sistema de salud, sistemas familiares^(25-26,28)^, patrones de vida, disponibilidad y adecuación de recursos^(25,28-29)^, factores ambientales, barreras y beneficios percibidos, conflicto de decisión, conocimiento insuficiente^(30)^, sentimiento de impotencia, susceptibilidad percibida^(31)^, demandas excesivas y dificultad para manejar un régimen de tratamiento complejo^(32)^.

El pictograma que expresa la relación entre los conceptos fue actualizado según las sugerencias propuestas, demostrando la interacción entre los conceptos de esta TSE (Figura 4).

**Figura 4 -:** Pictograma que involucra metaparadigmas, conceptos centrales y periféricos de la Teoría de la Situación Específica para el control de la salud en la insuficiencia cardíaca. São Paulo, Brasil, 2022

A pesar de la importancia de una buena gestión de la salud para la mejora de la calidad de vida y la reducción de hospitalizaciones, se sabe que muchos pacientes con IC presentan el diagnóstico de enfermería Control Ineficaz de la Salud. Diversos factores internos y externos al individuo influyen en la adquisición del control de la salud y, aunque no todos sean susceptibles de intervenciones de Enfermería, su identificación puede facilitar el proceso diagnóstico por parte del enfermero.

Al guiar al enfermero en la identificación tanto de los aspectos clínicos que indican que la enfermedad no está bajo control como de los factores ambientales e individuales que interfieren en la capacidad del individuo para controlar su salud adecuadamente, esta teoría busca contribuir a la adopción de estrategias que fomenten la inclusión del régimen terapéutico en la vida diaria del individuo con IC, mejorando su calidad de vida y su supervivencia.

Sus proposiciones reflejan las interacciones entre los conceptos establecidos y son susceptibles de futuras pruebas con la población en cuestión. Para ello, podrán utilizarse las actividades de Enfermería propuestas en las intervenciones de la NIC Cuidados cardíacos [4040], Educación en salud [5510] y Enseñanza: proceso de enfermedad [5602], así como los indicadores de los resultados NOC Autocontrol de la enfermedad cardíaca [1617] y Comportamiento de adherencia [1600].

## Conclusión

La validación realizada mediante la técnica Delphi permitió que la teoría fuera evaluada por especialistas con amplia experiencia, posibilitando la mejora de su texto y pictograma para reflejar de manera más fiel el fenómeno estudiado (control de la salud) y facilitar su incorporación en la práctica diaria de los enfermeros que atienden a individuos con IC, ya sea en un contexto ambulatorio o en el nivel de atención primaria. Además, el análisis realizado permitió confirmar que la teoría construida presenta las propiedades y características esperadas de una TSE.

Validar una teoría, ya sea desde una perspectiva interna o mediante su prueba empírica, no es una tarea sencilla y requiere, además de tiempo y dedicación, una gran experiencia en la construcción teórica y en la temática abordada. La técnica Delphi, aunque aún es poco utilizada con este propósito, ha demostrado ser útil al permitir la obtención de consenso sin la necesidad de un gran número de especialistas, lo que resulta particularmente ventajoso en áreas con escasez de expertos. Además de la dificultad de encontrar profesionales con la experiencia adecuada para la validación propuesta, un desafío importante fue el tiempo de respuesta de los jueces en cada ronda, lo que impactó en el tiempo total de conclusión del estudio.

Tras la validación interna y actualización, la teoría de situación específica para el control de la salud en IC ofrece una mejor comprensión de los factores que pueden influir en el control de la salud de los individuos con IC. Al estar fundamentada en la Teoría de Enfermería del Déficit del Autocuidado de Orem y utilizar conceptos provenientes de los lenguajes estandarizados NANDA, NIC y NOC, ampliamente aplicados en la práctica clínica, se espera que esta teoría contribuya a eliminar la dicotomía entre pensamiento y acción, permitiendo que el enfermero en la práctica pueda diagnosticar claramente los aspectos que involucran el control de la salud de sus pacientes y determinar la mejor intervención de Enfermería para estos individuos. Se espera que, en el futuro, se desarrollen instrumentos que permitan una evaluación más concreta del individuo y, para ello, podrán utilizarse los indicadores de los resultados NOC “comportamiento de adherencia” y “autocontrol de la enfermedad cardíaca” como guías.

Dado que la atención al individuo con insuficiencia cardíaca implica un enfoque multiprofesional y que algunos factores que influyen en el control de la salud no pueden ser abordados exclusivamente por la Enfermería, se considera que esta teoría también podría ser útil para otras disciplinas.
